# The Active Component of Aspirin, Salicylic Acid, Promotes *Staphylococcus aureus* Biofilm Formation in a PIA-dependent Manner

**DOI:** 10.3389/fmicb.2017.00004

**Published:** 2017-01-23

**Authors:** Cristian Dotto, Andrea Lombarte Serrat, Natalia Cattelan, María S. Barbagelata, Osvaldo M. Yantorno, Daniel O. Sordelli, Monika Ehling-Schulz, Tom Grunert, Fernanda R. Buzzola

**Affiliations:** ^1^Departamento de Microbiología, Parasitología e Inmunología, Facultad de Medicina, Instituto de Investigaciones en Microbiología y Parasitología Médica, Consejo Nacional de Investigaciones Científicas y Técnicas (CONICET), Universidad de Buenos AiresBuenos Aires, Argentina; ^2^Facultad de Ciencias Exactas, Centro de Investigación y Desarrollo de Fermentaciones Industriales (CINDEFI), Centro Científico Technológico Consejo Nacional de Investigaciones Científicas y Tócnicas (CTT CONICET La Plata), Universidad Nacional de La PlataLa Plata, Argentina; ^3^Functional Microbiology, Institute for Microbiology, University of Veterinary MedicineVienna, Austria

**Keywords:** biofilm, *Staphylococcus aureus*, salicylic acid, PIA, *codY*, iron, MRSA

## Abstract

Aspirin has provided clear benefits to human health. But salicylic acid (SAL) -the main aspirin biometabolite- exerts several effects on eukaryote and prokaryote cells. SAL can affect, for instance, the expression of *Staphylococcus aureus* virulence factors. SAL can also form complexes with iron cations and it has been shown that different iron chelating molecules diminished the formation of *S. aureus* biofilm. The aim of this study was to elucidate whether the iron content limitation caused by SAL can modify the *S. aureus* metabolism and/or metabolic regulators thus changing the expression of the main polysaccharides involved in biofilm formation. The exposure of biofilm to 2 mM SAL induced a 27% reduction in the intracellular free Fe^2+^ concentration compared with the controls. In addition, SAL depleted 23% of the available free Fe^2+^ cation in culture media. These moderate iron-limited conditions promoted an intensification of biofilms formed by strain Newman and by *S. aureus* clinical isolates related to the USA300 and USA100 clones. The slight decrease in iron bioavailability generated by SAL was enough to induce the increase of PIA expression in biofilms formed by methicillin-resistant as well as methicillin-sensitive *S. aureus* strains. *S. aureus* did not produce capsular polysaccharide (CP) when it was forming biofilms under any of the experimental conditions tested. Furthermore, SAL diminished aconitase activity and stimulated the lactic fermentation pathway in bacteria forming biofilms. The polysaccharide composition of *S. aureus* biofilms was examined and FTIR spectroscopic analysis revealed a clear impact of SAL in a *codY*-dependent manner. Moreover, SAL negatively affected *codY* transcription in mature biofilms thus relieving the CodY repression of the *ica* operon. Treatment of mice with SAL induced a significant increase of *S aureus* colonization. It is suggested that the elevated PIA expression induced by SAL might be responsible for the high nasal colonization observed in mice. SAL-induced biofilms may contribute to *S. aureus* infection persistence in vegetarian individuals as well as in patients that frequently consume aspirin.

## Introduction

*Staphylococcus aureus* is a common commensal of the human nostrils (Kaspar et al., 2016). While asymptomatic colonization of *S. aureus* does not necessarily lead to illness, the loss of the mucosal or epithelial surface integrity can be responsible for *S. aureus* diverse diseases (Fitzpatrick et al., 2005). Persistent and difficult-to-eradicate infections can be caused by both methicillin-susceptible *S. aureus* (MSSA) (Lattar et al., 2012) and methicillin-resistant *S. aureus* (MRSA).

Changes in nutrient availability and the presence of distinct molecules during the infectious process can be detected by *S. aureus*, which quickly modifies the expression of metabolic, regulatory and virulence genes thus adapting to life in a dynamic environment (Somerville and Proctor, 2009). One of the strategies used by *S. aureus* to respond to unfavorable conditions is the formation of biofilm, which plays a key role in chronic persistent infections, such as osteomyelitis and foreign body-related infections (Archer et al., 2011). It has also been suggested that biofilms would be responsible, in part, for late reactivation of staphylococcal chronic infections after the initial disease was healed (Ciampolini and Harding, 2000; Brady et al., 2008). Biofilms are a complex aggregation of bacteria commonly encased into an adhesive matrix composed of extracellular substances. In *S. aureus*, the extracellular matrix is composed of proteins, DNA and the polysaccharide intercellular adhesin (PIA). The amount of these matrix components may vary according to the surrounding environmental conditions (Furukawa et al., 2006). The mechanism of biofilm formation is classified as PIA-independent or PIA-dependent, when PIA is the major component of the extracellular matrix (Otto, 2013). PIA is coded by the *icaADBC* operon and synthesized from UDP-*N*-acetylglucosamine during the exponential growth phase (Otto, 2013). Conversely, the capsular polysaccharide (CP) encoded by the *cap* operon is predominantly produced during the post-exponential growth phase, even though it is synthesized from the same biosynthetic precursor (O'Riordan and Lee, 2004). In addition, the PIA and CP synthesis depends upon the tricarboxylic acid (TCA) cycle and the expression of both polysaccharides is opposite in iron-deficient conditions (Vuong et al., 2005; Sadykov et al., 2010a). At this point it is worth mentioning that the TCA cycle is regulated by Fur (ferric uptake regulator) in an iron-dependent manner and by CodY, among other transcriptional factors (Richardson et al., 2015).

CodY, a metabolite-responsive global regulator, controls metabolism and virulence gene expression through several molecular mechanisms (Richardson et al., 2015). CodY represses *ica* and also *cap* transcripts (Majerczyk et al., 2010) in *S. aureus* (Richardson et al., 2015). Disruption of the *codY* gene in a *S. aureus* clinical isolate that strongly produces biofilm resulted in very low PIA production and showed reduction in biofilm formation (Tu Quoc et al., 2007). Similarly, deletion of the *codY* gene in *S. aureus* USA300 resulted in increased production of secreted proteases which negatively modified biofilm formation (Rivera et al., 2012). In contrast, another study showed that *codY* mutants of two *S. aureus* clinical isolates, SA564 and UAMS-1, displayed high capacity to form biofilms, apparently resulting from elevated levels of *ica* transcripts and PIA accumulation (Majerczyk et al., 2008). Notably, CodY is positively affected by iron (Friedman et al., 2006). Modifications of the intracellular iron concentration may then alter the activity of CodY. In fact, several TCA cycle enzymes utilize iron in the form of iron-sulfur clusters and, therefore, iron depleted growth conditions diminish the TCA cycle activity drastically (Varghese et al., 2003).

Salicylic acid (SAL) is a small molecule derived from plants with pleiotropic effects on eukaryote and prokaryote cells (Price et al., 2000; Patrignani and Dovizio, 2015). In addition, SAL is the main biometabolite of aspirin, the popular nonsteroidal anti-inflammatory agent regularly utilized by millions of individuals worldwide due to its known analgesic and cardiovascular protective activities. Furthermore, vegetarian individuals contain similar plasma concentrations of SAL when compared with patients consuming low daily doses of aspirin (Rajaram, 2003). The expression of virulence factors and regulatory genes is modified by SAL in several bacterial species (Pomposiello et al., 2001; Denkin et al., 2005). Previous findings from our laboratory demonstrated that exposure of encapsulated *S. aureus* strains to low concentrations of SAL reduced CP production and increased the Eap adhesin expression under planktonic conditions (Alvarez et al., 2010, 2011). On the other hand, Johnson et al. (2008) observed that an increase of Eap expression under depleted iron conditions contributed to biofilm formation, a finding that becomes relevant due to the fact that SAL can form complexes with iron cations (Cheng et al., 1996; Pozdnyakov et al., 2015).

This study was designed to elucidate whether the iron content limitation provoked by SAL can modify the metabolism and/or metabolic regulators thus leading to an altered expression of PIA and CP by *S. aureus* adopting the biofilm lifestyle. The comprehension of the effects of SAL on *S. aureus* biofilm formation would permit to understand how this pathogen adapts to a moderate iron-limited environment as well as to design better therapeutic approaches to combat adaptation of *S. aureus* to the host and chronic infection. The outcome of early *S. aureus* infection in a host who takes aspirin daily or feeds on a vegetarian diet only may be different from that expected in an otherwise healthy host.

## Materials and methods

### Bacterial strains and growth conditions

*S. aureus* laboratory strain Newman and the isogenic derivatives *codY* (Newman Δ*codY*::*ermC*) (Luong et al., 2011) and *ica* (Newman *ica*::*tet*) (Kropec et al., 2005) mutants were used in this study. In addition, 8 clinical isolates related to USA300 and USA100 clones (Table 1), which were identified previously by *spa* typing, MLST and *SCCmec* typing (Lattar et al., 2012) were included in the study. *S. aureus* SA113 and Reynolds CP5 null (Δ*cap5*) (Watts et al., 2005) strains were also included as reference controls. Bacteria were stored in Trypticase Soy Broth (TSB) (Britania, Buenos Aires, Argentina) with 20 % glycerol at −80°C until use. All cultures were grown in TSB supplemented with 0.25% of glucose (TSBg) medium in the presence or absence of 2 mM salicylic acid (SAL) with or without 50 μM FeSO_4_ and incubated for 24 h at 37°C and 200 r.p.m. The iron addition to TSBg did not affect the biofilm formation by *S. aureus* (Figure S1). Iron-restricted conditions were assessed in iron-depleted TSBg medium (CTSBg) by batch incubation with 3% w/v Chelex 100 (BioRad, Hercules, CA, USA). For selection of the chromosomal marker in the Newman *ica* and *codY* mutants 5 μg/ml tetracycline and 10 μg/ml erythromycin were used, respectively. All chemical reagents were purchased from Sigma-Aldrich (St. Louis, MO, USA) unless otherwise indicated.

**Table 1:** **Genomic features of ***S. aureus*** clinical isolates**.

**Strain**	**Spa type**	**MLST**		**SCCmec**	**Course of infection**
		**CC**	**ST**		
CBS	t149	5	5	I	–
BRZ	t138	8	239	II	–
AR48	t149	5	5	I	Chronic
AR66	t002	5	100	IV	Acute
AR94	t008	8	8	IV	Chronic
AR56	t149	5	5	–	Chronic
AR70	t002	5	5	–	Acute
AR83	t002	5	5	–	Chronic
Newman	t008	8	8	–	–

*S. aureus strains related to USA300 (CC8) or USA100 (CC5) clones were chosen. CBS and BRZ strains are representatives of the Cordobés and the Brazilian HA-MRSA clones, respectively. S. aureus AR isolates come from different hospitals of Buenos Aires City, Argentina. Multilocus sequence types (MLST), clonal complexes (CC) and sequence types (ST) were determined previously. The defining feature of MRSA is the presence of the staphylococcal cassette chromosome mec (SCCmec). Different SCCmec types were established by specific PCR assays. The spa types from sequences of the protein A (spa) repeat region were assessed from the Ridom spa server (http://spa.ridom.de) (Lattar et al., 2012)*.

### Chelating capacity of SAL

The chelating capacity of SAL was assessed by the ferrozine assay (Mladenka et al., 2011). Briefly, TSBg medium containing different concentrations of SAL (final volume: 100 μl) was supplemented with 50 μM FeSO_4_ and incubated for 5 min. To avoid iron oxidation, 50 μl of 5 mM NH_2_OH solution was added to the mixture. Then, 50 μl of 5 mM ferrozine (or distilled water for the blanks) was added. The iron-ferrozine complex formation was determined by determination of the absorbance at 540 nm (Abs_540_).

### Intracellular iron contents in biofilms

Aliquots of bacterial lysates generated by incubation of biofilms over 24 h with lysis buffer [100 μl of buffer Tris-EDTA 10:1, (10 mM Tris-HCl pH 8; 1 mM EDTA), 40 μl of 1 mg/ml lysostaphin, 20 μl of 50 mg/ml lysozyme] were treated with HCl/KMnO_4_ (a solution of equal volumes of 1.4 M HCl and 4.5 w/v KMnO_4_ in distilled H_2_O) to promote the release of iron from the bacterial proteins. The Fe^2+^ contents were determined by the ferrozine assay and normalized to the protein concentration on each sample determined by the Bradford method (Bradford, 1976).

### Biofilm formation assay

Quantitative assessment of biofilm formation was performed as previously described with modifications (Trotonda et al., 2008). Briefly, *S. aureus* strains were grown for 18 h and diluted 1:100 in TSBg or TSBg with SAL and FeSO_4_, when required. Two hundred μl of these cell suspensions were added to sterile 96-well polystyrene microtiter plates. Non-inoculated medium controls were included. After 24 h of static incubation at 37°C, the final culture density (named OD_G_) was measured by reading the optical density (OD_595_) using a microplate reader (Multiskan EX, Thermo Electron Corporation, Waltham, MA, USA). The culture medium was then removed from each well and plates were washed twice with phosphate buffered saline (PBS). The biofilms were fixed with 100% methanol for 15 min, stained with 0.5% crystal violet for 20 min, and washed twice again gently under running tap water. The amount of biofilm biomass was measured after addition of 30% glacial acetic acid by reading the OD_595_ (named OD_B_). The levels of crystal violet staining were expressed relative to the final culture density measured prior to the biofilm assay (biofilm: OD_B_)/OD_G_) and named in the text as “biofilm” for the sake of clarity. The induction of biofilm by SAL was defined as the mean value from SAL-treated biofilms relative to the mean value of the biofilm formed in TSBg. The relative amount of biofilm formed by each isolate to that of the SAL-treated Newman biofilm was expressed as a percentage. For detachment assays, biofilms were grown for 24 h in 96-well microplates as described above. The biofilms were washed with PBS and then treated for 2 h at 37°C with 20 μg/ml of Dispersin B (Kane Biotech Inc., Winnipeg, MB, Canada) in PBS (Trotonda et al., 2008). After treatment, the biofilms were washed with PBS, fixed with methanol, stained with crystal violet and quantified as described above.

### Biofilm visualization by microscopy

Overnight cultures of *S. aureus* grown in TSBg, TSBg with SAL or TSBg with SAL plus iron were adjusted to an OD_600_ of 0.05 and aliquots were utilized to inoculate the wells of a 24-well plate (scanning electron microscopy, SEM) or a 8-well chambered coverglass (ThermoFisher Scientific, Waltham, MA, USA) (confocal laser scanning microscopy, CLSM). After static incubation during 24 h at 37°C the biofilms were processed as follows. For SEM, biofilms were washed with PBS, fixed with 2.5% formaldehyde for 2 h at 4°C and dehydrated in increasing concentrations of ethanol. The glass coverslips were fixed on aluminum stubs, covered with gold-palladium film and examined in a Philips XL30 TMP scanning electron microscope. For CLSM, after washing with PBS, the biofilms were stained using the LIVE/DEAD BacLight Bacterial Viability kit (Molecular Probes, Waltham, MA, USA) to determine bacterial viability, or treated with 90 μg/ml wheat germ agglutinin (WGA) (Oregon Green® 488 conjugate, Molecular Probes) with 5 μg/ml FM 4-64 (Molecular Probes) for PIA visualization, incubated for 15 min in the dark and fixed with 4% paraformaldehyde. After adding PBS, the biofilms were visualized with a Leica confocal laser scanning microscope (model TCS SP5, Germany). With the mixture of the SYTO9® and propidium iodide stains (LIVE/DEAD BactLight Bacterial Viability kit's fluorophores), bacteria with intact cell membranes display green fluorescence, whereas bacteria with damaged membranes exhibit red fluorescence. SYTO9 and propidium iodide were excited at 498–565 nm and their emission was monitored at 600–693 nm. Oregon Green 488® and FM 4-64 were excited at 508–549 nm and their emission was monitored at 650–750 nm. In all cases, 0.7 μm optical sections from the entire biofilm were collected and the stacks of images were analyzed using the Leica LAS AF Lite software. The biofilm parameters were determined using the Comstat 2 software. Images from 2 ramdonly selected positions of 2 independent samples were analyzed.

### Fourier transform infrared (FTIR) spectroscopy analysis

*S. aureus* biofilms were grown in 25-cm^2^ polystyrene tissue culture flasks at 37°C statically for 24 h in TSBg or TSBg with SAL and FeSO_4_, when required. The biofilms were scraped, suspended in PBS and centrifuged at 8000 × g for 20 min. Planktonic cultures grown up to stationary phase were washed three times in PBS and sedimented by centrifugation. Aliquots of the pellets suspended in deionized water were spotted on a zinc selenite (ZnSe) optical plate and dried during 40 min to yield transparent films. These films were used directly for FTIR spectroscopy, which was conducted with an HTS-XT microplate adapter coupled to a Tensor 27 FTIR spectrometer (Bruker Optics GmbH, Ettlingen, Germany). Infrared spectra were recorded in transmission mode in the spectral range between 4000 and 500 cm^−1^. Normalized second-derivative spectra of the spectral window from 1200 to 800 cm^−1^ were selected for principal component analysis (PCA) using the Unscrambler X software (CAMO Software, Oslo, Norway). This spectral region is dominated by C-O-C and C-O-P stretching vibrations of various oligosaccharides and polysaccharides and their specific types of glycosidic linkages and was previously shown to recognize changes in *S. aureus* surface glycopolymer composition including CP (Grunert et al., 2013; Johler et al., 2016).

### Quantification of PIA and CP in biofilms

*S. aureus* biofilms were grown in 96-well plates as described above. After measuring the OD_595_ of the cultures (OD_G_), the biofilms were washed twice with PBS and fixed with methanol. Then, the quantity of PIA and CP was determined by ELISA or by a fluorometric assay as specified below.

CP from *S. aureus* Newman (serotype 5; CP5) biofilms was quantified by ELISA. Briefly, 100 μl of blocking solution [1%, w/v, BSA in PBST (PBS+0.05% Tween 20)] was added to fixed biofilms and incubated for 1h at 37°C. After removing the blocking solution 100 μl of CP5 antiserum was added (1:3000 in PBST) and the plate was incubated for 30 min at 37°C. After washing with PBST the plate was dried and 100 μl of protein A-horseradish peroxidase conjugate (HRP-protein A Invitrogen, Carlsbad, CA, USA) (1:3000 in PBST) was added and incubated for 30 min at 37°C. The wells were washed three times for 5 min in PBST. The substrate [1 ml of 10 mg/ml *o*-phenylenediamine plus 9 ml of citrate (0.1 M Na_2_HPO_4_ pH = 5) plus 10 μl of H_2_O_2_ 30V] was then added, the plate was incubated for 5 min at 37°C. The reaction was stopped by addition of 50 μl of H_2_SO_4_ 12.5% per well and the level of CP expressed was measured by the absorbance at 492 nm (Abs_492_) relative to OD_G_.

The PIA produced in biofilms formed by the CBS strain (Table 1) was quantified by ELISA. The procedures were similar to those described above except that, after blocking solution removal, 100 μl per well was added of 75 ng/ml wheat germ agglutinin (WGA)-HRP conjugate, a lectin that binds to PIA sugar residues. The Abs_492_ was measured and related to OD_G_. The PIA produced in biofilms formed by the Newman and the BRZ (Table 1) strains was quantified by fluorometry using 100 μl per well of 90 μg/ml WGA-Oregon Green 488® conjugate (Molecular Probes). After 15 min of incubation of the plate in the dark, excess amounts of stain were removed and wells were washed twice with PBS. After adding 100 μl of PBS per well, the fluorescence was measured with a Fluorometer FLx800 (Biotek Instruments Inc., Winooski, VT, USA). Oregon Green was excited at 485 nm and its emission was detected at 528 nm. The level of PIA was expressed relative to the final culture density measured (PIA F/OD_G_). For all assays, four independent experiments were performed in sixtuplicate.

### RNA extraction from biofilm

*S. aureus* biofilms were grown in 75-cm^2^ polystyrene tissue (T75) culture flasks at 37°C statically for 6 or 24 h in TSBg or TSBg with SAL and FeSO_4_, when required. Flasks were chosen for culture in order to make available a large surface area to support biofilm growth. After static incubation for 6 or 24 h the supernatants were removed from each flask. Biofilm cells were scraped, transferred to an Eppendorf tube and enzymatically disrupted (100 μl of buffer Tris-EDTA 10:1, 40 μl of 1 mg/ml lysostaphin, 20 μl of 50 mg/ml lysozyme). Then, the lysates were exposed to Trizol Reagent® (Invitrogen) and the bacterial RNA extracted according to the manufacturer's protocol.

### Real-time quantitative reverse transcription PCR (qRT-PCR)

Bacterial RNA from 6 to 24 h biofilms was obtained as described above. After DNAse treatment using the RQ1 RNAse free DNAse (Promega, Madison, WI, USA), cDNA synthesis was performed with the ImProm-II™ Reverse Transcriptase kit (Promega). qRT-PCR was performed using the HOT FIREPolEvaGreen® qPCR Mix Plus (ROX) (Solis Biodyne, Tartu, Estonia) and Applied Biosystems 7500 instrumental, using the primers detailed in Supplemental material. The *gyrB* gene was used to normalize data. The number of copies of each sample transcript was determined with the aid of the 7500 system SDS software (Applied Biosystems, Carlsbad, CA, USA). The 2^−ΔΔCt^ value represents the difference in threshold cycle (Ct) between the target and control (*gyrB*) genes treated with SAL or SAL with Fe^2+^, minus the difference in Ct between untreated (TSBg) target and control genes (Alvarez et al., 2010).

### Protein extracts and SDS-PAGE

Protein extracts were prepared from *S. aureus* biofilm grown in T75 culture flasks containing 40 ml of TSBg in the presence or absence of SAL at 37°C under static conditions for 24 h. The biofilms were scrapped and centrifuged at 10,000 × g for 20 min. Pellets were suspended in 0.6 ml of lysis buffer [30% raffinose in 0.05 M Tris (pH7.5) with 0.145 M NaCl] containing 100 μg/ml lysostaphin and 1 mM PMSF (phenyl-methyl-sulfonil-fluoride) and incubated 1 h at 37°C at 200 r.p.m. The suspension was centrifuged twice at 8000 × g for 15 min at 4°C and supernatants were saved for analysis. The protein concentration of each sample was measured using the Bradford method. An equal volume of 6 × Laemmli sample buffer was added to the protein extracts prior to boiling them for 3 min and separating them by SDS-10% PAGE. SDS-PAGEs were stained with Coomassie blue.

### Mass spectrometric analysis of proteins

Selected bands were excised from the SDS-polyacrylamide gel. After destaining, the samples were reduced, alkylated and hydrolyzed with 25 ng/μl of trypsin. The samples were analyzed with a MALDI-TOF spectrometer, Ultraflex II (Bruker Daltonics, Billerca, MA, USA) at the Mass Spectrometry Facility (CEQUIBIEM, Buenos Aires, Argentina). The criteria for protein identification were based on the manufacturer's definitions. The protein score is −10 × log *p*, where *p* is the probability that the observed match is a random event. Protein scores higher than 81 were considered significant (*p* < 0.05).

### Aconitase activity assay

Aconitase activity was quantified according to the method described by Kennedy et al. (1983). Biofilm of Newman strain grown during 24 h with or without 2 mM of SAL or SAL plus 50 μM of FeSO_4_, were suspended in PBS and centrifuged at 9300 × g for 5 min and suspended in lysis buffer containing 90 mMTris-HCl (pH 8.0), 100 μM fluorocitrate and 100 μg/ml lysostaphin. The suspensions were incubated for 15 min at 37°C and centrifuged at 21,000 × g for 30 min at 4°C. A 20 μl aliquot of the cell lysate was added to 180 μl assay buffer [100 mM Tris-HCl (pH 8.0), 50 mM trisodium citrate] and incubated for 5 min at 37°C. The amount of aconitate produced was quantified by measuring the Abs_240_. A molar absorption coefficient of 3.6 mM^−1^ cm^−1^ was used and 1 U aconitase was defined as the enzyme activity that catalyzes the formation of 1 μm aconitate per min. Protein concentrations were determined by the Bradford assay.

### Concentration of lactate, glucose and acetate in biofilm supernatants

Supernatants from biofilms grown for 24 h were recovered by centrifugation. The concentration of lactate, glucose and acetate were determined using kits purchased from Sigma-Aldrich and following the manufacturer‘s indications. The index of lactate (IL) production by glucose consumption was defined as IL = nm lactate_TSBg_/mg glucose_TSBg_. The lactate concentration produced by biofilms treated with SAL or SAL plus FeSO_4_ was determined as: [lactate]_Treated_ = IL × [glucose]_Treated_.

### pH measurement and transferrin-Fe release

pH was measured using an Adwa AD12 pH meter (Szeged, Hungary) on supernatants from *S. aureus* cultured up to exponential phase in TSBg with or without SAL. The iron released from human transferrin was determined as previously described (Friedman et al., 2006). The transferrin-Fe complex concentration was measured at Abs_450_ every 1 min for 15 min upon addition of 40 μM human transferrin to all samples.

### Nasal colonization murine model

Eight-week-old male CF1 outbred mice weighing ~25 g were obtained and maintained at the vivarium of the Instituto de Investigaciones en Microbiología y Parasitología Médica (Universidad de Buenos Aires-CONICET) in accordance with the international guidelines set forth. The experimental procedures were evaluated by the Institutional Animal Care and Use Committee (CICUAL) and approved by resolution N° 901/16 of the School of Medicine, University of Buenos Aires. Thirty minutes before the bacterial challenge, mice randomly split into 2 groups of 5–6 animals received by the intravenous route 200 μl of 2 mM SAL or PBS (control), respectively. Then, a suspension containing an inoculum of 10 μl of 1.6 × 10^7^ CFU of the *S. aureus* Newman strain was pipetted slowly into the mouse nares. After 4 h, mice were euthanized with CO_2_. The area around the nasal region was wiped out with 70% ethanol, and the nose was excised and homogenized in 400 μl TSB using a tissue grinder. Dilutions of the tissue homogenate were plated onto TSA plates for CFU counting (Barbagelata et al., 2011).

### Statistical analysis

Nonparametric data were analyzed with the Mann-Whitney test. Data with normal distribution were compared with the paired *t*-test, using the Graphpad Software (version 6.0; GraphPad Prism). *P*-values < 0.05 were considered significant.

## Results

### SAL decreases the free iron content

**Figure 1:**
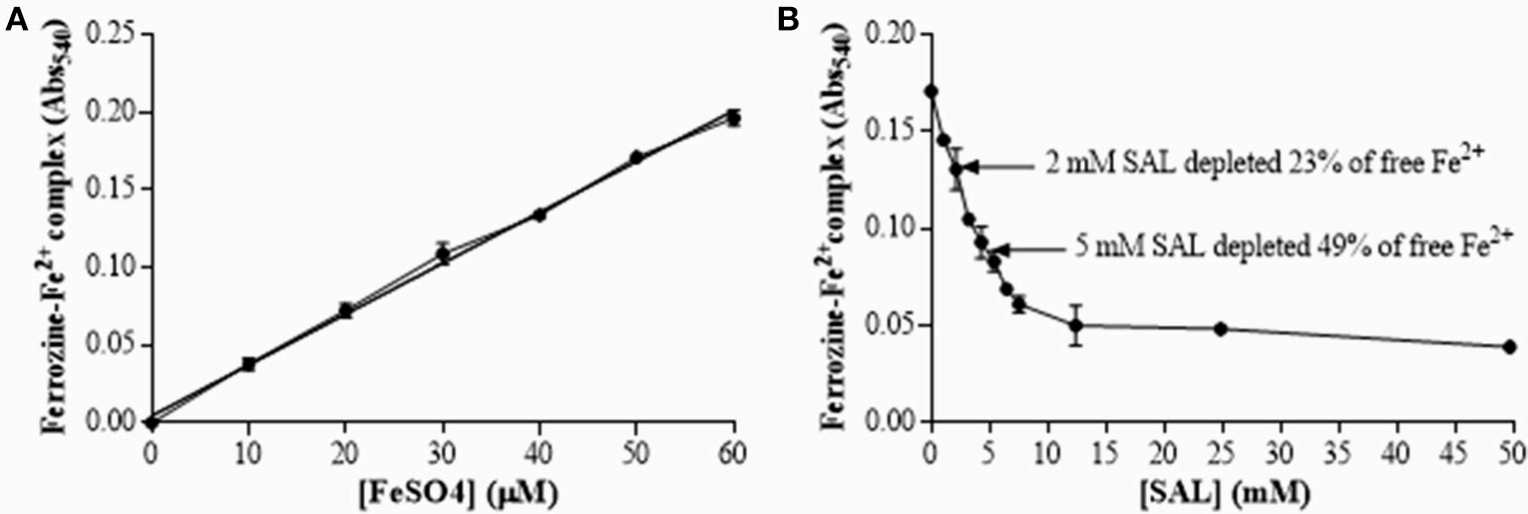
**SAL chelating capacity of iron. (A)** Standard curve of ferrous ion concentration in ferrozine complexes determined in TSBg medium using 1.25 mM ferrozine. **(B)** Ferrozine-Fe^2+^ complex quantification in TSBg treated with different concentrations of SAL mixed with 50 μM FeSO_4_. The amount of ferrozine-Fe^2+^ complex was determined at an absorbance of 540 nm (Abs_540_). Basal iron concentration in TSBg was 39.3 μM.

**Figure 2:**
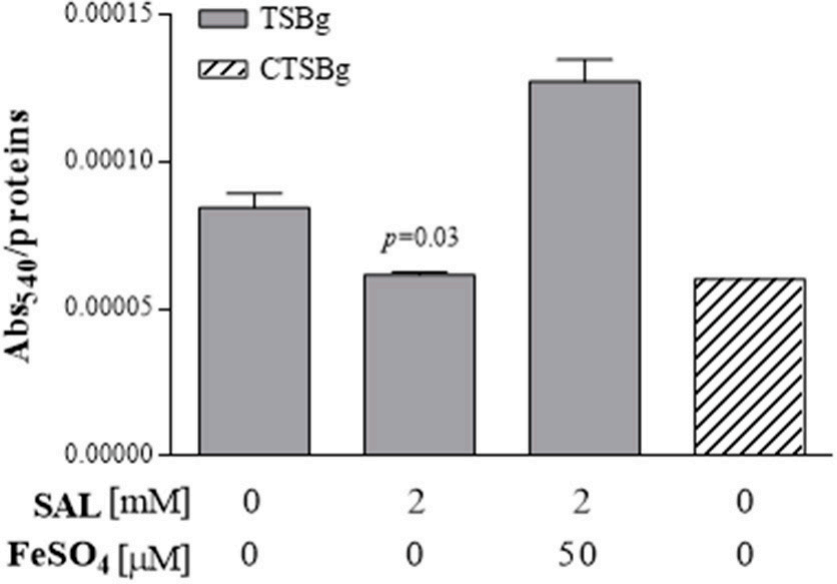
**Intracellular ferrous ion contents after SAL treatment of ***S. aureus*** biofilms**. Each bar represents the arithmetic mean ± SEM of Abs_540_ related to μg/ml of protein/well of samples measured in triplicate from 3 independent experiments. Comparison of SAL-treated vs. untreated groups was significantly different (*p* = 0.03) (Mann-Whitney test). CTSBg (TSBg treated with Chelex-100) was utilized as reference value.

Ferrozine assays were conducted to determine the SAL chelating capacity in TSBg medium. As shown the Figure 1, a depletion of 23% of free Fe^2+^ was observed at a concentration of 2 mM of SAL. Increased concentrations of SAL provoked a constant decrease (~80%) of available free Fe^2+^ reaching a plateau at ~12.5 mM. Although 5 mM of SAL depleted the 49% of free Fe^2+^ ions, the concentration of 2 mM of SAL was chosen in this study because this is the serum concentration normally achieved after ingestion of aspirin at doses within the therapeutic range. Furthermore, the concentration of 5 mM produces toxic effects. Similar results were obtained by measuring the total free iron present in TSBg (39.3 ± 7.1 μM) and TSBg with 5 mM of SAL (21.4 ± 5.3 μM) by atomic absorption spectroscopy (Analytical Chemistry Facility, School of Biochemistry and Pharmacy, Univ. Buenos Aires). Interestingly, the intracellular free Fe^2+^ concentration in *S. aureus* forming biofilms when grown in TSBg with 2 mM of SAL was 27% lower than that obtained in the control without SAL addition, and similar to that observed in TSBg treated with Chelex-100 (Figure 2). Therefore, 2 mM of SAL decreases the free iron content at a similar rate in both intracellular and extracellular environments.

### SAL enhances formation of biofilm by MSSA and MRSA strains

**Figure 3:**
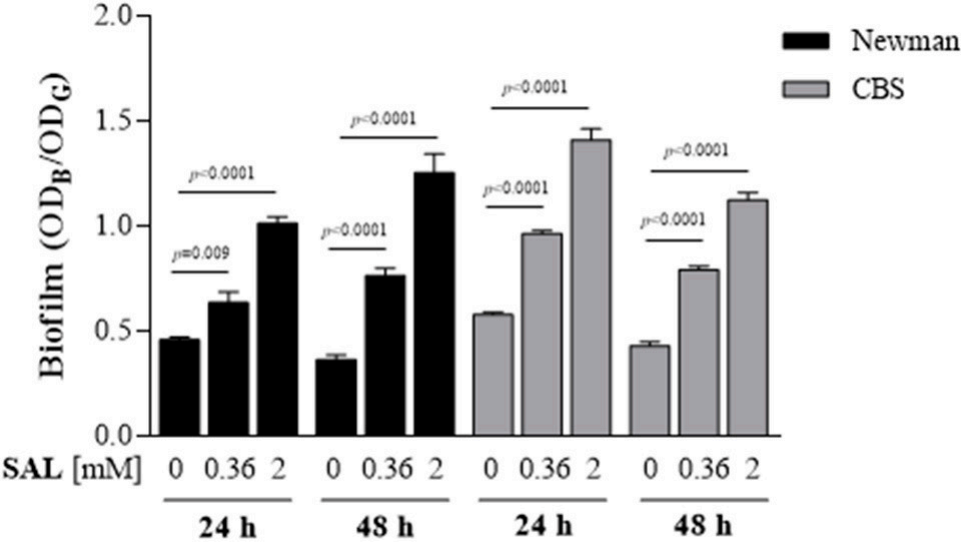
**Biofilm formation by the Newman and CBS strains exposed to SAL**. Static cultures treated with two different concentration of SAL were analyzed after 24 and 48 h and biofilm formation by the Newman and CBS strains was compared. Each bar represents the arithmetic mean ± SEM of 6–8 wells from 3 to 4 independent experiments. Biofilm formation values correspond to the OD_595_ of crystal violet (OD_B_) measured relative to the final culture density (OD_G_) after 24 or 48 h incubation. Comparisons are represented by lines and each *p*-value is denoted above (Mann-Whitney test).

**Figure 4:**
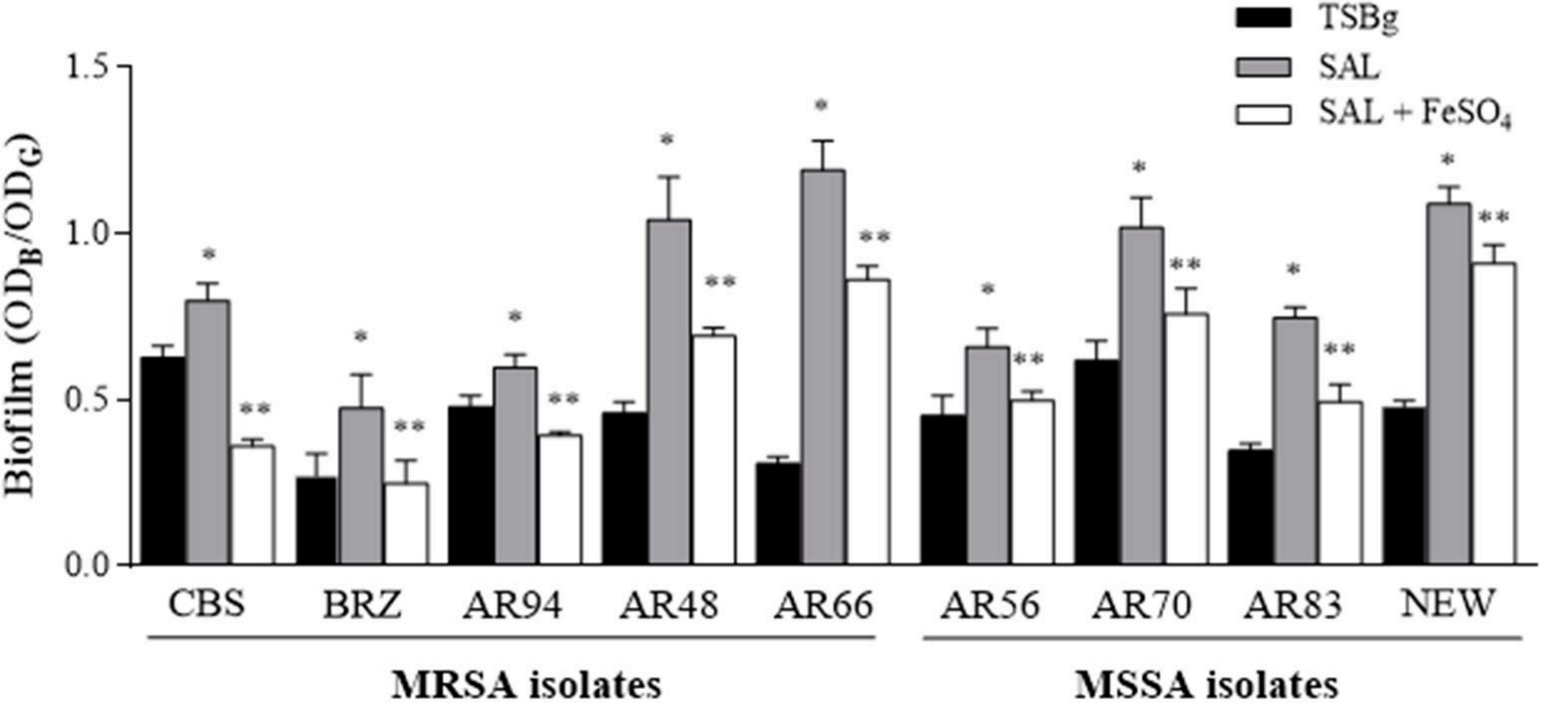
**The effect of SAL on biofilm formed by MRSA and MSSA strains**. Biofilms were formed in TSBg during 24 h in the presence or absence of 2 mM of SAL or SAL plus 50 μM of FeSO_4_. Each bar represents the arithmetic mean ± SEM of 6 wells from 4 independent experiments. The biofilms were quantified by crystal violet staining (OD_B_) and expressed relative to the final culture density (OD_G_). Statistically significant differences were represented by asterisks: (^*^) SAL-treated group vs. untreated group; (^**^) SAL-treated group vs. SAL plus iron-treated group. *p* < 0.05 were considered significant (Mann-Whitney test).

**Figure 5:**
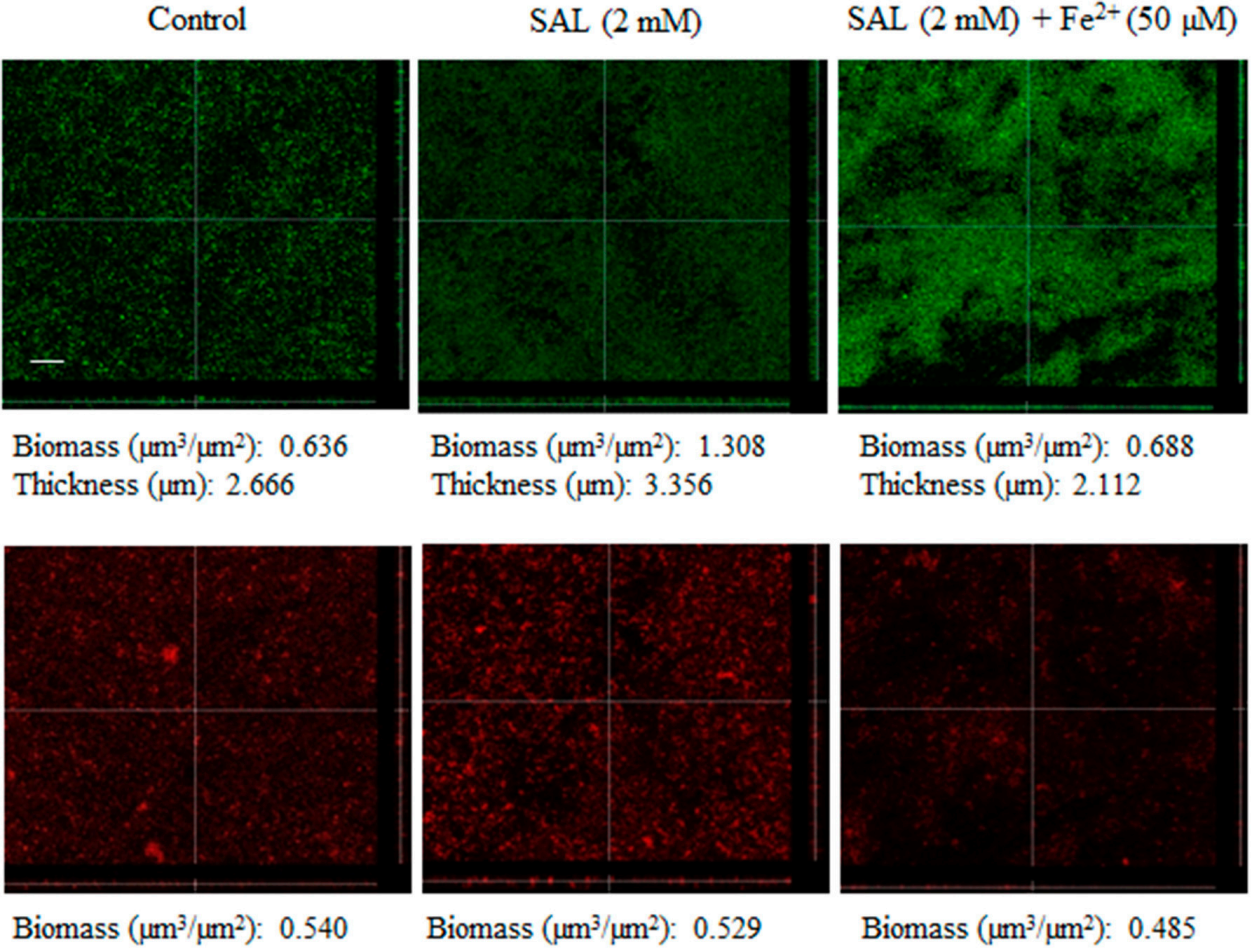
**Confocal laser scanning microscopy (CLSM) images of Newman biofilms exposed to SAL or SAL plus iron**. Biofilms formed in TSBg with or without SAL or SAL plus iron during 24 h were stained with LIVE/DEAD BacLight stain. Upper and bottom panels show green (live) and red (dead) fluorescing cells, respectively. Sagittal sections of the biofilms are shown below and to the right of each panel. Scale bar: 10 μm. Results are representative of two experiments. By means of COMSTAT2 software analyses of images the maximum thickness (μm) of biofilms and the biomass were determined. Biomass is the amount of biologic material -in volume- present in a given area (μm^3^ of sample per μm^2^ of covered glass surface).

Initially, Newman and CBS (Table 1) strains were chosen to be treated with different SAL concentrations to establish the biofilm formation at various growth times (Figure 3). The biofilms of both strains grown in the presence of 0.36 or 2 mM of SAL showed a significant increase at both time points (24 or 48 h) when compared with those observed in the control groups. Then, *S. aureus* clinical isolates related to the USA300 and USA100 clones (Table 1) were selected to determine the chelating effect of SAL on biofilm formation since different strains may respond dissimilarly to available iron. Both the MRSA and the MSSA strains showed increased biofilm production under 2 mM SAL exposure whereas iron addition provoked a significant reduction of the biomass (Figure 4). The values of SAL-biofilm induction ranged from 1.25 (AR94) to 3.87 (AR66) for the strains studied. Values of the biomass relative to the Newman strain treated with SAL were as follows: CBS (73%), BRZ (43%), AR94 (54%), AR48 (95%), AR66 (109%), AR56 (60%), AR70 (93%), and AR83 (68%). It should be noted that addition of iron to TSBg did not affect the biofilm formation by *S. aureus* (Figure S1). Furthermore, the biofilms formed by the Newman strain in the presence of SAL were stained with the LIVE/DEAD BacLight Bacterial Viability kit and visualized by CLSM. The COMSTAT analyses of images from live bacteria with intact cell membranes acquiring green fluorescence established that exposure to 2 mM of SAL provoked the increment in the biomass (1.308 μm^3^/μm^2^) and the biofilm maximum thickness (3.356 μm) when these were compared with the controls (biomass: 0.636 μm^3^/μm^2^ and maximum thickness: 2.666 μm). Addition of iron to SAL containing medium diminished both the biomass (0.688 μm^3^/μm^2^) and biofilm maximum thickness (2.112 μm) (Figure 5). The dead-bacteria (red fluorescence) biomass values were similar among the samples studied (control: 0.540 μm^3^/μm^2^; SAL: 0.529 μm^3^/μm^2^; SAL plus Fe^2+^: 0.485 μm^3^/μm^2^). The MTT colorimetric assay was used to quantify the viable bacteria in biofilms following each treatment. As shown in Figure S2, SAL and SAL plus iron treatments did not modify the levels of metabolically active bacterial cells forming biofilm. Taken together all these results suggest that diminution of [Fe^2+^] caused by SAL induced an increment of biofilm formation by *S. aureus* regardless of the methicillin susceptibility or clonal genomic characteristics.

### SAL affects the polysaccharide composition in biofilms examined by FTIR spectral analyses

**Figure 6:**
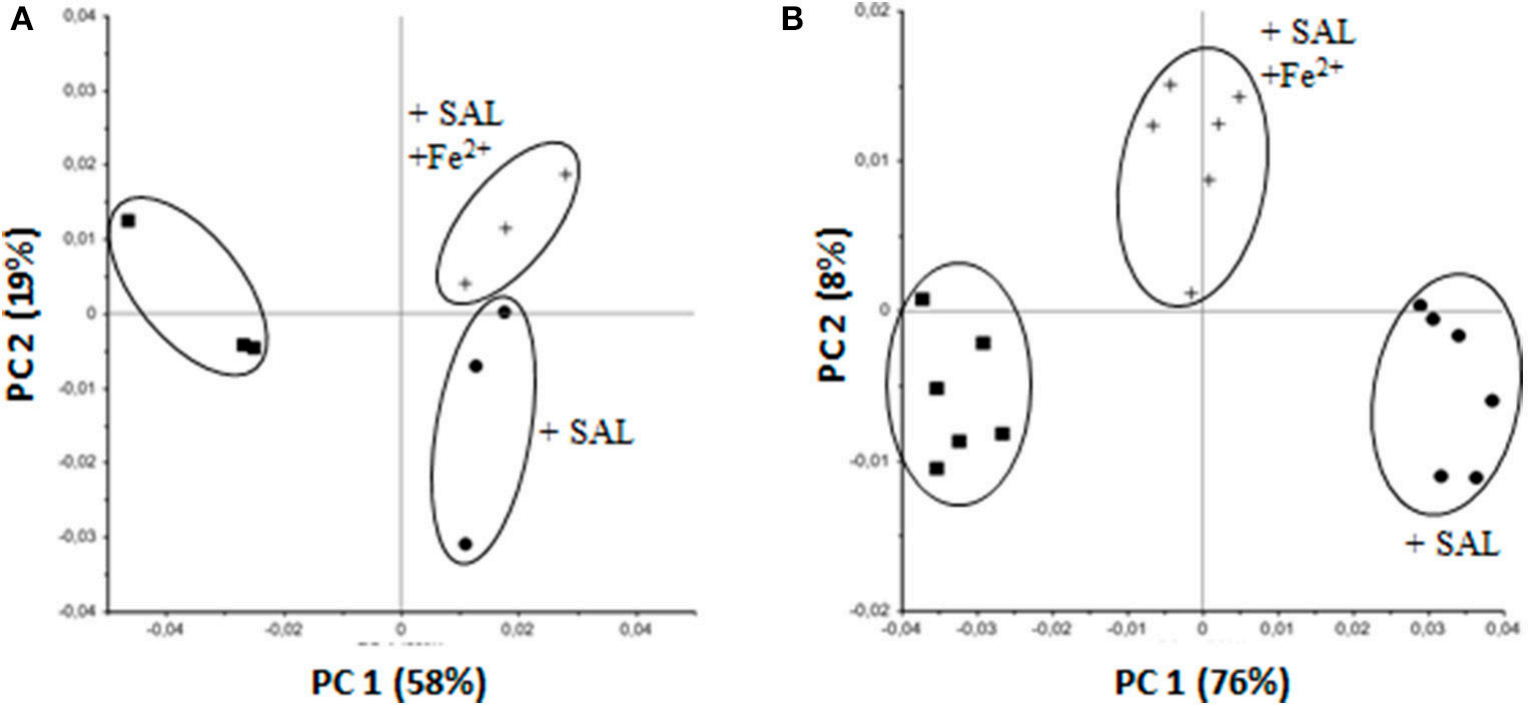
**Impact of SAL and SAL plus iron on ***S. aureus*** monitored by FTIR spectroscopy**. PCA was carried out on second derivative, vector-normalized FTIR spectra in the spectral range for glycopolymers (1200–800 cm^−1^). **(A)** Newman strain, planktonic culture. **(B)** Biofilm formed by the Newman strain. In both cases bacteria were grown in TSBg with or without 2 mM of SAL or SAL plus 50 μM FeSO_4_. Clusters are indicated with ellipses. Symbols are defined in the figure. The relative contribution of each principal component is indicated in parentheses.

**Figure 7:**
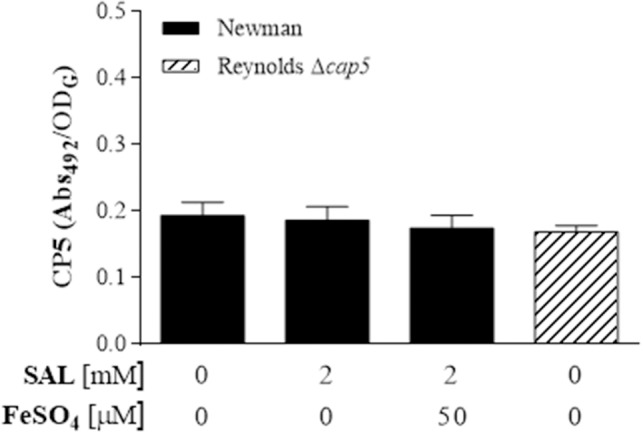
**Capsular polysaccharide (CP) expression in biofilm formed by the Newman strain**. Biofilms were developed in static cultures for 24 h at 37°C in TSBg supplemented with SAL or FeSO_4_ as indicated. CP serotype 5 was assessed by ELISA. Each bar represents the arithmetic mean ± SEM of the Abs_492_ relative to the final culture density (OD_G_). Negative control: *S. aureus* Reynolds CP5-null.

*S. aureus* synthesize mainly two polysaccharides, CP and PIA. In our previous work it was demonstrated that CP expression is reduced when planktonic *S. aureus* is grown in the presence of low concentrations of SAL (Alvarez et al., 2010). Thus, to establish the effect of iron chelation by SAL on *S. aureus* surface polysaccharides, chemometric-assisted FTIR spectroscopic measurements were performed from *S. aureus* planktonic cultures and biofilms. The resulting FTIR spectra provided highly specific molecular fingerprints derived from stretching and bending vibrations of all functional groups of the bacterial cell or biofilm matrix (Helm et al., 1991). Based on their specific biochemical constituents, FTIR spectra can be subdivided into several spectral partitions including fatty acid and phosphorus-containing biomolecules of membrane components (e.g., phospholipids), proteins of the bacterial cell and surface-associated polysaccharides. The latter spectral window (1200–800 cm^−1^) was used for the PCA, an unsupervised multivariate statistical method, to investigate the discriminatory features of the SAL treatment on *S. aureus* polysaccharide production. The score plot revealed a clear clustering of spectral data according to the treatment conditions (untreated, SAL-treated and SAL plus Fe^2+^). As shown in Figure 6A, SAL treatment of planktonic cultures of the Newman strain caused a perturbation of the surface polysaccharide composition that was not reverted by iron addition. In the light of our previous work and the results described herein it is suggested that CP instead of PIA expression is affected by SAL treatment due to the fact that CP production under planktonic culture conditions is limited (Alvarez et al., 2010). In contrast to planktonic growth, PCA of the Newman biofilms revealed that iron limitation by SAL exposure induced polysaccharide production alterations that were partially reverted by iron addition (Figure 6B). Indeed, ~25% decrease of [Fe^2+^] by SAL chelation is enough to alter the expression of polysaccharides when *S. aureus* is forming biofilm. In fact, *S. aureus* did not produce CP5 when bacteria are forming biofilms in any of the conditions investigated (Figure 7). *S. aureus* CP5-deficient Reynolds strain was utilized as negative control of CP expression. Taken together the results suggest that the polysaccharide perturbations induced by SAL in biofilms -which occurred mainly in PIA rather than in CP5 and in potentially other glycopolymers structures (e.g., LTA, WTA)- might be affected by SAL under both growth conditions.

### SAL induces PIA production by modifying the bacterial metabolic status of biofilm

**Figure 8:**
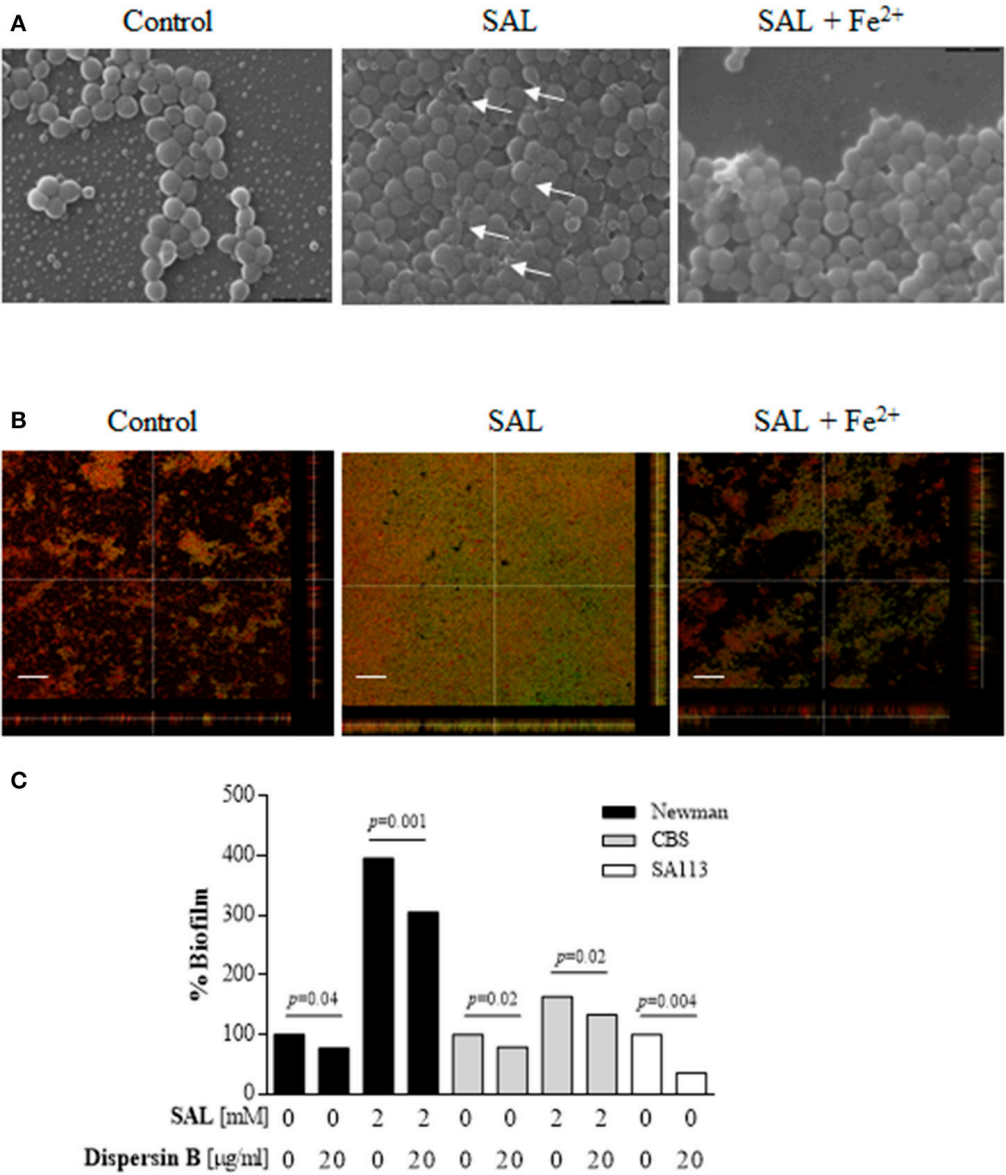
**Assessment of extracellular polysaccharide matrix in the Newman strain biofilms. (A)** Scanning electron microscopy (SEM) of Newman biofilms grown statically for 24 h at 37°C in TSBg supplemented with 2 mM of SAL or 50 μM of FeSO_4_ as indicated. The magnification is 10,000 ×. Arrows show extracellular substances between adjacent cells. **(B)** Visualization of the extracellular polysaccharide matrix in Newman biofilms by CLSM after staining with green fluorescent labeled WGA. Bacterial cells were stained with the lipophilic membrane red dye FM 4-64. Sagittal sections of the biofilms are shown below and to the right of each panel. Scale bar: 10 μm. Results are representative of two experiments. **(C)** The effect of Dispersin B treatment on the detachment of the Newman biofilms. Each bar represents the percentage of biofilm formed after the treatments. Lines indicate statistically significant decrease of biofilm formation by cells attached following Dispersin B treatment compared with the untreated biofilms.

**Figure 9:**
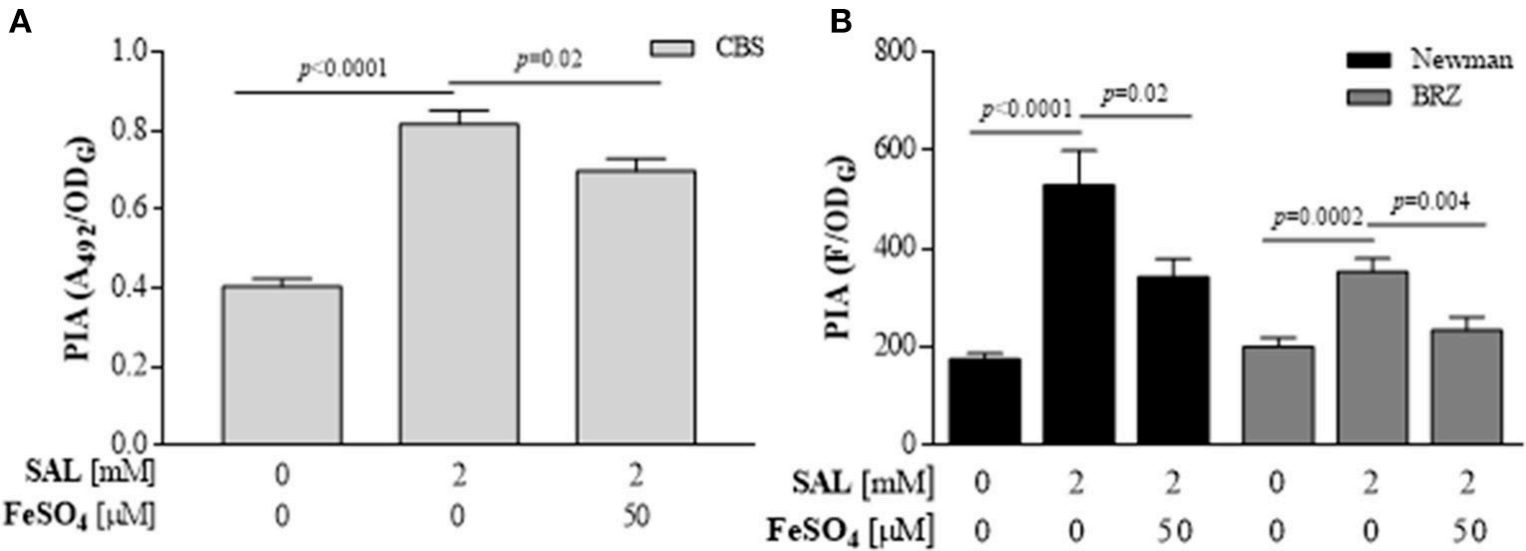
**Quantification of PIA produced in SAL-induced ***S. aureus*** biofilms. (A)** PIA produced by CBS strain assessed by ELISA assay. **(B)** PIA expresses by BRZ and Newman strains assessed by the fluorometric assay. Each bar represents the mean ± SEM of quadruplicate measurements of from 3 independent experiments. The values of Abs_492_ or fluorescence (F) were relative to the final density of culture (OD_G_). Comparisons are represented by lines and each *p*-value is denoted above.

*S. aureus* biofilm formation in the presence of SAL was visualized by SEM. Biofilm images revealed that SAL caused more aggregation of Newman cells and that these cells produced more extracellular substances compared with the cells in the control biofilm, which exhibited distinct images of bacteria with sharp contours. The addition of iron to the culture medium with SAL diminished the enclosing material of the bacterial cells (Figure 8A). To gain additional insight into the surface polysaccharides affected by exposure to SAL, the PIA from biofilms was visualized by CLSM after staining with green fluorescent labeled WGA and the red lipophilic membrane dye. As shown in Figure 8B, according to the high contents of green (PIA) and yellow staining (PIA and cell colocalization), SAL exposure of Newman biofilms would induce the PIA production. Interestingly, addition of iron to the culture medium containing SAL resulted in less green and yellow staining sectors suggesting the presence of minor contents of PIA in the extracellular matrices. Moreover, in the presence of iron, the biomasses induced by SAL did not differ from those of the control. To support these evidences *S. aureus* biofilms were treated with Dispersin B, a β-hexosaminidase that degrades carbohydrates as the PIA. The *S. aureus* SA113 strain was utilized as reference because PIA is the major extracellular component when this strain adopts the biofilm lifestyle (Di Poto et al., 2009). The enzymatic treatment provoked a significant detachment of the Newman and CBS biofilms grown in the presence of SAL (Figure 8C). Furthermore, the quantity of PIA expressed in the biofilms by Newman, BRZ and CBS strains increased with SAL exposure and significantly diminished with iron addition (Figure 9). These results indicate that exposure to SAL induced PIA-dependent biofilm, which is formed by both MSSA and MRSA strains.

**Figure 10:**
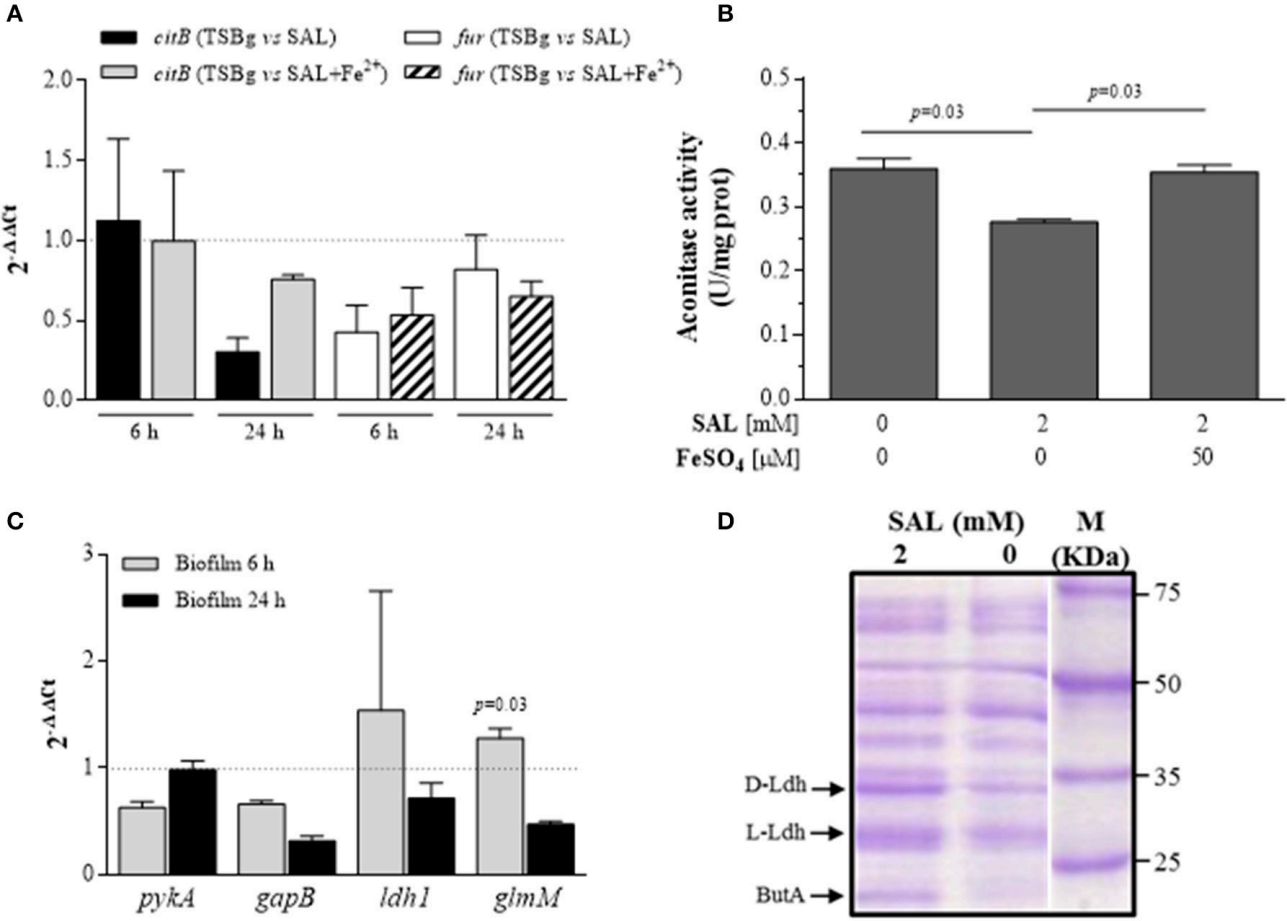
**Metabolic status of ***S. aureus*** biofilms treated with SAL. (A)** Expression of *citB* and *fur* transcripts from immature (6 h) and mature (24 h) biofilms formed by the Newman strain in the presence of 2 mM of SAL or SAL plus 50 μM of FeSO_4_. Changes in gene expression are shown as normalized mean fold change [2^(−ΔΔCt)^] ± SEM (differences of target gene expression with SAL or SAL+Fe^2+^ compared with target gene expression in TSBg). Data were normalized to *gyrB* expression. Untreated biofilms (TSBg groups) were used as controls (controls = 1, represented by a dotted horizontal line). 2^(−ΔΔCt)^ >1 represents significant increased expression and 2^(−ΔΔCt)^ < 1 indicates significant decreased expression. Each bar represents the arithmetic mean of duplicate measurements from 3 independent experiments. **(B)** Aconitase activity quantification in biofilms produced by Newman strain grown during 24 h in TSBg supplemented with SAL or FeSO_4_ as indicated. The amount of aconitase produced was related to mg of total proteins. Each bar represents the arithmetic mean ± SEM of 3 independent experiments in triplicate. **(C)** Expression of *pykA, gapB, ldh1*, and *glmM* transcripts from Newman biofilms grown during 6 or 24 h in the presence of 2 mM of SAL or SAL plus 50 μM of FeSO_4_. Changes in gene expression are shown as normalized mean fold change [2^(−ΔΔCt)^] ± SEM (differences of target gene expression with SAL or SAL+Fe^2+^ compared with target gene expression in TSBg). Data were normalized to *gyrB* expression. Untreated biofilms (TSBg groups) were used as controls (controls = 1, represented by a dotted horizontal line). **(D)** Cell wall cellular protein profiles assessed by SDS-PAGE. Bands represent proteins extracted from Newman biofilms cultured during 24 h in TSBg supplemented with SAL or FeSO_4_ as indicated. Abbreviations are: D- and L-Ldh (D- and L-lactate dehidrogenase, respectively) and ButA (acetoin reductase). Equivalent volumes of cell extracts were loaded into each lane. Arrows indicate the polypeptides confirmed in each fraction by MS/MS (MALDI-TOF).

Iron limitation has deep impact on the bacterial metabolism. Since certain enzymes of the TCA cycle, such as aconitase (*citB*), contain iron-sulfur binding clusters, and because decreased TCA activity in *S. epidermidis* is associated with high levels of PIA expression (Vuong et al., 2005), it can be speculated that the elevated PIA production observed in *S. aureus* biofilms treated with SAL may be associated to reduced TCA activity due to the intracellular iron limitation induced by SAL. Initially, the analyses at the transcriptional level showed that SAL decreased the transcription of *citB* in mature (24 h) biofilms and the relative *citB* expression increased slightly by addition of iron (Figure 10A). Indeed, the fold change of *fur* expression was low in the presence of SAL and did not suffer modifications by iron addition in the biofilms grown during 6 or 24 h (Figure 10A). The *citB* gene expression data were reinforced by functional assays. Aconitase enzymatic activity was measured in biofilms grown on the conditions under study during 24 h. As shown the Figure 10B, SAL exposure significantly decreased the aconitase activity and the addition of iron restored the activity of the enzyme to levels similar to the control. Therefore, the results obtained suggest that iron limitation by SAL negatively affects the activity of the TCA cycle of bacteria forming biofilm.

**Figure 11:**
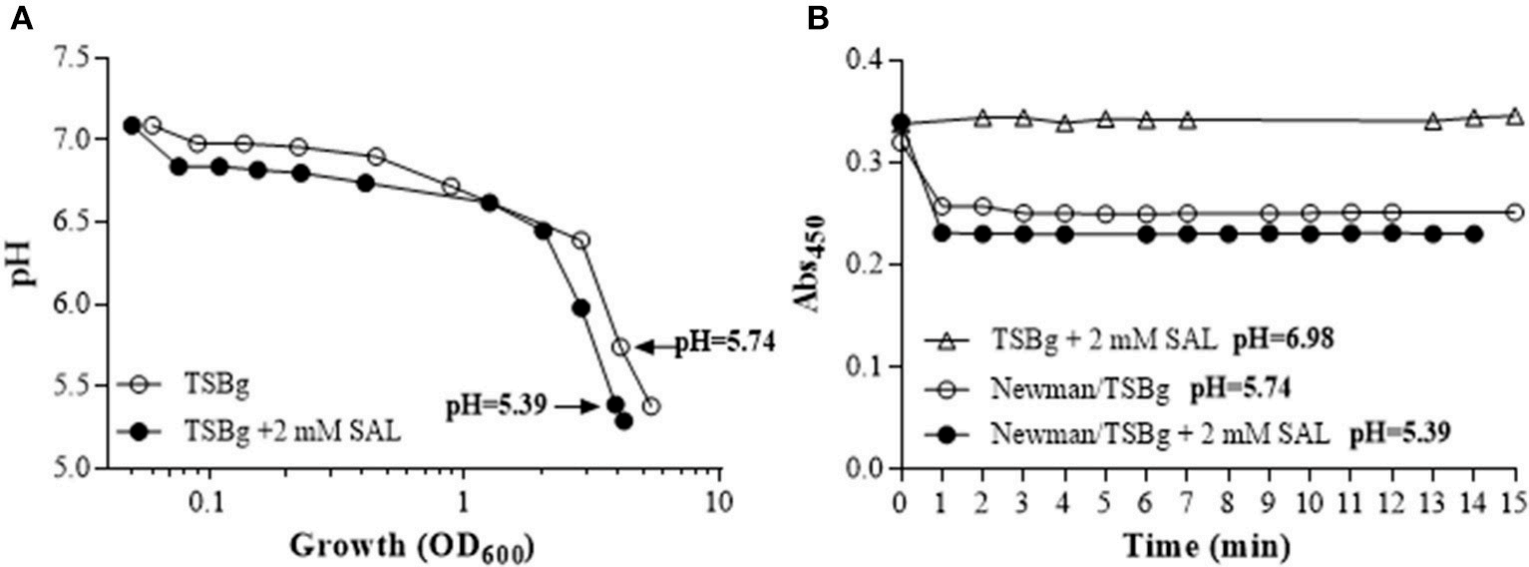
**SAL decreases extracellular pH during ***S. aureus*** growth favoring iron release from human transferrin. (A)** Curves depict extracellular pH vs. growth (OD_600_) of the Newman strain exposed or not to 2 mM of SAL. **(B)** Spectrophotometric assessment of the release of iron bound to human transferrin in late-exponential cultures (OD_600_ = 4, see arrows on **A**) with or without SAL. Graphics are representative of three independent experiments.

Since the production of a polysaccharide requires high expenditure of energy and the SAL diminished the TCA cycle activity, we hypothesized that the fermentative pathway would be preferential in bacteria forming biofilm in the presence of SAL. Total RNA extracted from immature and mature biofilms grown in the presence or absence of SAL was utilized to assess the transcriptional levels of enzyme indicators of glycolysis (pyruvate kinase gene *pykA*), gluconeogenesis (glyceraldehyde-3-phosphate dehydrogenase gene *gapB*), lactic fermentation (lactate dehydrogenase 1 gene *ldh1*), and UDP-glucosamine precursor (phosphoglucosamine mutase gene *glmM*) by qRT-PCR assays. Indeed, the relative level of *glmM* expression was increased significantly by SAL in immature biofilms (fold change: 1.275 ± 0.09) when compared with that of mature biofilms (fold change: 0.471 ± 0.02) (Figure 10C). The presence of SAL did not increase significantly the transcription of any of the other genes studied. Moreover, SAL reinforced the down-fold change of *gapB* expression in mature biofilms (Figure 10C). Figure 10D depicts the SDS-PAGE profile of cell wall proteins extracted from mature biofilms treated or not treated with SAL. The results revealed that certain bands were intensified by SAL treatment of biofilm formed by the Newman strain. Each one of these bands were analyzed by MS/MS (MALDI-TOF) and the proteins L- and D-lactate dehydrogenases (Ldh) (score: 134 and 253, respectively; *p* < 0.05) and acetoin reductase (ButA) (score: 86, *p* < 0.05) were identified. The acetate, glucose and L-lactate concentrations in supernatants of mature biofilms were also evaluated. There was no acetate accumulation in supernatants of biofilms grown during over 24 h in any of the experimental conditions under study (data not shown). Since 2 mM of SAL causes a slight growth delay of the Newman strain in TSBg (Figure S3) and, consequently, the glucose consumption was diminished, the index of L-lactate (IL) production over glucose consumption (IL = nmol lactate_TSBg_/mg glucose_TSBg_) in biofilms formed during 24 h was determined. The IL of the Newman strain biofilm was 855 nmol/mg. The amount of L-lactate produced by the Newman strain biofilm treated with SAL was high ([lactate]_SAL_ = 855 nmol/mg × [glucose]_SAL_ = 1393.6 nmol) whereas iron addition slightly diminished it ([lactate]_SAL+Fe_ = 855 nmol/mg × [glucose]_SAL+Fe_ = 1308 nmol). Finally, SAL exposure of *S. aureus* during growth diminished significantly the extracellular pH values (*p* < 0.01, *t*-test correct for multiple comparisons using Holm-Sidak method) thus promoting the release of iron from human transferrin (Figure 11). The results suggest that SAL only stimulates the lactic fermentation pathway since the acetate levels were undetectable and the Ldh expression was enhanced by SAL.

### SAL negatively affects *codY* transcription

**Figure 12:**
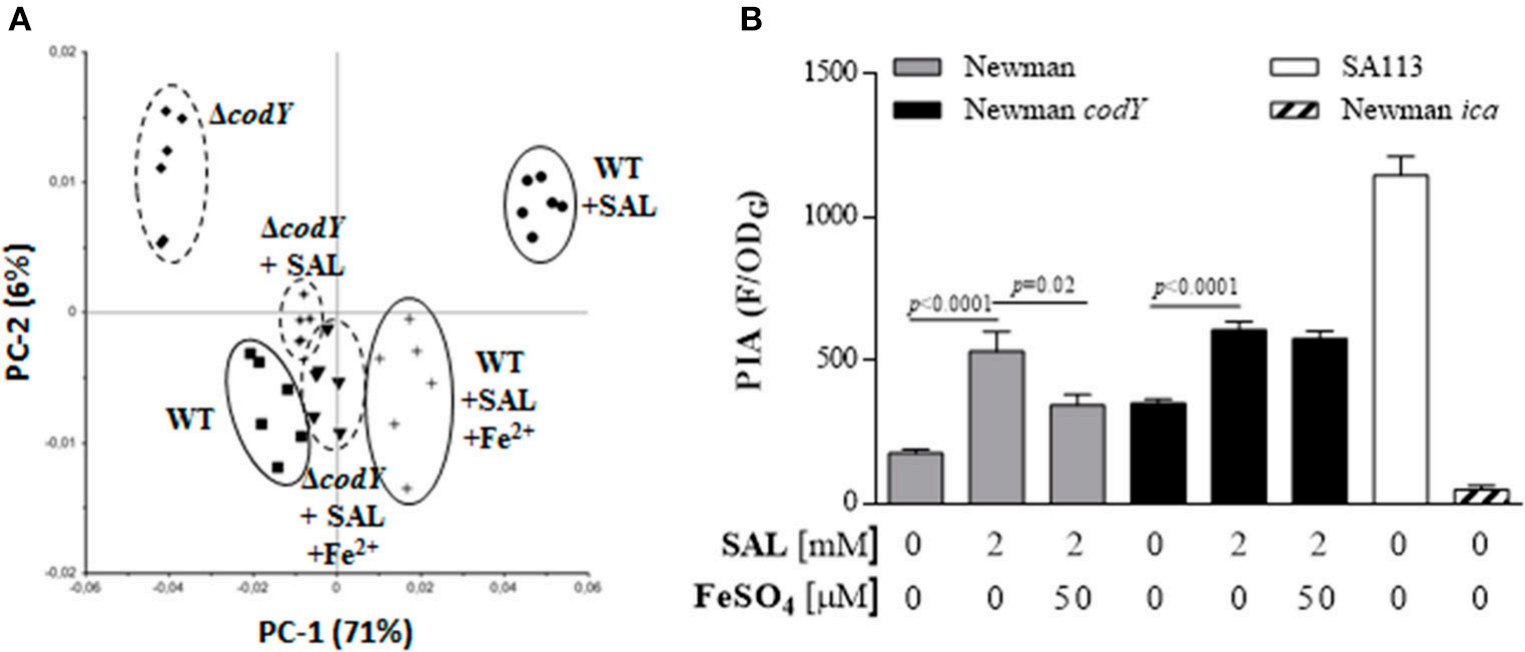
**Interplay of ***codY*** and SAL in biofilms. (A)** The surface glycopolymer composition was examined by FTIR spectroscopy. PCA was carried out on second derivative, vector-normalized FTIR spectra in the spectral range of 1200–800 cm^−1^ derived from biofilm produced by the strain Newman and its isogenic *codY* mutant. Bacteria were grown in TSBg in the presence or absence of 2 mM of SAL with or without 50 μM of FeSO_4_. Clusters are indicated with ellipses. Symbols are defined in the figure. The relative contribution of each PC is indicated in parentheses. **(B)** PIA production by the Newman *codY* mutant in biofilms was assessed by fluorometric assays. Each bar represents the arithmetic mean ± SEM of quadruplicate measurements from 3 independent experiments. The values of fluorescence (F) were related to the OD of growth (OD_G_) in each well. Comparisons are represented by lines and *p*-values are denoted above. Data from the SA113 and Newman *ica*-deficient mutants were included as positive and negative controls, respectively.

The main *S. aureus* polysaccharides coded by the *cap* and *ica* operons can be repressed by CodY (Majerczyk et al., 2008, 2010; Thoendel et al., 2011). Hence, the effect of SAL on CodY was investigated. To this purpose, biofilms produced by the Newman *codY* deficient mutant grown in the presence of SAL or SAL plus iron were subjected to FTIR spectroscopic analysis. As shown in Figure 12A, the PCA revealed remarkable differences of qualitative and quantitative spectral features in the polysaccharide region (1200–800 cm^−1^) between the wild-type, the *codY* mutant and the wild-type grown in the presence of SAL. Interestingly, the effect of *codY* on the surface polysaccharide composition was partially reversed by SAL as well as SAL plus iron. This finding was confirmed by assessment of spectral distance values, a quantitative measure of dissimilarity corresponding to the non-overlapping areas of the spectra derived from the wild-type strain. These values were: (i) wild-type (SAL), 0.76; (ii) wild-type (SAL + Fe^2+^), 0.35; (iii) *codY* mutant, 0.35; (iv) *codY* mutant (SAL), 0.18; and (v) *codY* mutant (SAL+ Fe^2+^), 0.21. Therefore, *S. aureus* surface polysaccharides were affected by SAL in a *codY*-dependent manner. The amount of PIA expressed in the *codY* mutant biofilms significantly increased with SAL exposure as shown in Figure 12B. Furthermore, the SAL-induced increase of the PIA produced by *codY* mutant biofilms (1.7-fold) was lower than that observed in the biofilms formed by Newman (3-fold). When iron was added to the culture medium containing SAL, the level of PIA expressed by the *codY* mutant biofilms was not significantly different when compared with that determined in culture medium with SAL and without iron. Conversely, the amount of PIA in Newman biofilms formed in the presence of SAL was diminished by iron addition (Figure 12B).

**Figure 13:**
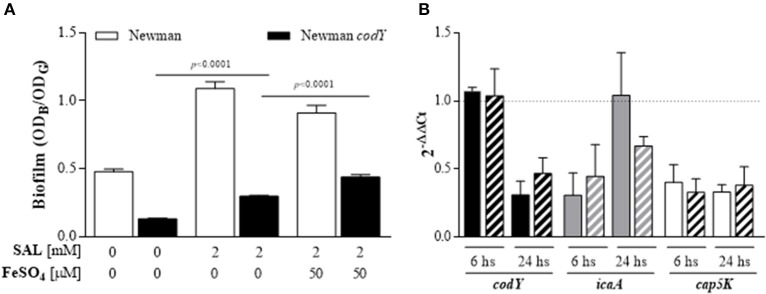
**SAL decreases the ***codY*** transcript level and CodY contributes to diminishing SAL-induced biofilm after iron addition. (A)** Biofilms formed by the Newman strain and its isogenic *codY* mutant in TSBg during 24 h in the presence or absence of 2 mM of SAL with or without 50 μM of FeSO_4_. Each bar represents the arithmetic mean ± SEM of sixtuplicate measurement from 3 independent experiments. The biofilms were quantified by crystal violet staining (OD_B_) and expressed relative to the final culture density (OD_G_). Comparisons are represented by lines and *p*-values are indicated above. **(B)** Expression of *codY, icaA*, and *cap5K* transcripts from immature (6 h) and mature (24 h) biofilms formed by the Newman strain grown in the presence or absence of 2 mM of SAL with or without addition of 50 μM of FeSO_4_. Changes in gene expression are shown as normalized mean fold change [2^(−ΔΔCt)^] ± SEM (differences of target gene expression with SAL or SAL+Fe^2+^ compared to target gene expression in TSBg). Data were normalized to *gyrB* expression. Untreated biofilms (TSBg groups) were used as controls (controls = 1, represented by dotted horizontal line). Plain bars: TSBg vs. SAL. Striped bars: TSBg vs. SAL+Fe^2+^. Each bar represents the arithmetic mean ± SEM of 3 independent experiments in duplicate.

The role of CodY on the SAL-induced biofilm formation was evaluated. In spite of the low biofilm formation by the *codY* mutant when compared with the parental strain, SAL treatment increased significantly the amount of biofilm formed by the *codY* mutant (Figure 13A). In contrast to the effect observed in the Newman strain, addition of iron to SAL-containing medium increased the biofilm production by the *codY* mutant when compared with that of biofilm grown in the presence of SAL, without iron (Figure 13A).

In order to study the interplay between *codY* and SAL, the levels of *codY, icaA*, and *capK5* transcripts from immature (6 h) and mature (24 h) biofilms formed by the Newman strain were evaluated by qRT-PCR (Figure 13B). As expected, SAL exposure decreased the transcription of *cap5K* in biofilms growing at 6 and 24 h and iron addition did not revert these decreases. On the other hand, the presence of SAL in mature biofilms relieved the decrease of *icaA* expression when biofilms were grown with SAL during 6 h. Consistent with this finding, the relative *codY* expression was significantly reduced in mature biofilms exposed to SAL. When iron was added, the decrease of *icaA* and *codY* expression in mature biofilms remained below the control values. Therefore, SAL exposure seems to relieve CodY repression of *ica* by diminishing *codY* transcript levels in mature biofilms.

### *S. aureus* nasal colonization increases with SAL treatment

**Figure 14:**
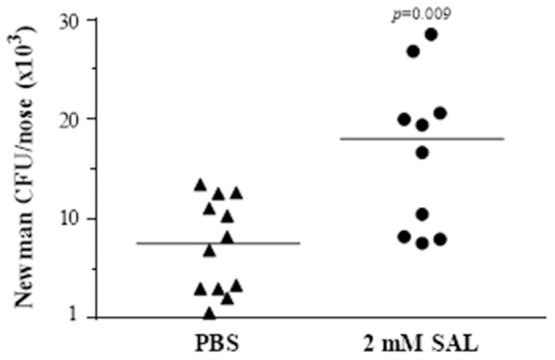
**SAL increases ***S. aureus*** murine nasal colonization**. Randomized groups of 5–6 mice were inoculated by the iv route with 2 mM of SAL or PBS 30 min before bacterial inoculation (1.6 × 10^7^ CFU/nose) of the Newman strain. The colonization status was determined following nasal excision at 4 h. Each dot represents an individual mouse. The horizontal lines represent the median value (PBS: 7.5 × 10^3^ CFU/nose; SAL: 1.8 × 10^4^ CFU/nose). Comparison among groups was significant (*p* = 0.009) (Mann-Whitney test).

The initial step in biofilm formation is the adhesion of bacteria to cells or inert surfaces. Because formation of *S. aureus* biofilm was demonstrated *in vivo* in both human and murine nasal mucosa (Zernotti et al., 2010; Muthukrishnan et al., 2011; Reddinger et al., 2016) and since PIA contributes significantly to the adherence of *S. aureus* to nasal epithelial cells (Lin et al., 2015) we investigated the effect of SAL *in vivo* using a nasal colonization murine model. Groups of mice received 200 μl of 2 mM of SAL or PBS (control) by the intravenous route 30 min before bacterial inoculum. The animals were then inoculated by the intranasal route with 10 μl of a suspension containing ~1.6 × 10^7^ CFU of the *S. aureus* Newman strain. Mice were sacrificed at 4 h after intranasal inoculation and the number of CFU in the nostrils was determined. The CFU number recovered from SAL-treated mice (median: 1.8 × 10^4^ CFU/nose) was significantly higher than that of the control group (median: 7.5 × 10^3^ CFU/nose) (*p* = 0.009, Mann-Whitney test) (Figure 14). These results show that SAL treatment induced a significant increase of *S. aureus* nasal colonization in mice.

## Discussion

Millions of individuals regularly take aspirin to reduce the risk of cardiovascular disease. Once ingested, aspirin is rapidly converted into SAL, the metabolite responsible for the known anti-thrombotic, analgesic and anti-inflammatory properties. Fruits and vegetables contain SAL, which plays a role in plant immunity, and the serum concentration of SAL in vegetarians overlaps with that of aspirin users (Blacklock et al., 2001). Many pleiotropic effects are exerted by SAL on plants, humans and bacteria. Indeed, the production of bacterial virulence factors is affected by the presence of SAL (Price et al., 2000). A peculiarity of this small molecule is its ability to form complexes with iron in aqueous solutions (Nichela et al., 2015). SAL is a weak acid that has the ability to pass through the cell membrane and form complexes with intracellular iron. The different mechanisms utilized to acquire iron and the importance of the expression of iron-regulated genes to pathogenesis underscores the essentiality of this nutrient to *S. aureus*. In the present study, we found that SAL not only decreased the free iron load in the culture medium but also caused moderate iron starvation in the intracellular milieu of *S. aureus* cells forming biofilms. As a consequence of the iron level diminution, *S. aureus* increased the biofilm biomass through a PIA-dependent mechanism. Moreover, SAL exhibited the ability to negatively alter the amount of *codY* transcripts, a negative regulator of the *ica* locus, in *S. aureus* cells forming biofilm.

Contradictory results were reported about the participation of iron and biofilm production in different bacterial species. In this regard, exposure to iron stimulated the biofilm formation by *Escherichia coli* (DePas et al., 2013) and *Pseudomonas aeruginosa* (Bomberger et al., 2016). Notably, films of the salicylic acid-releasing polymers were found to inhibit biofilm formation by *E. coli* or *P. aeruginosa* (Nowatzki et al., 2012). Although the mechanisms involved in this inhibition remain unproven, it can be speculated that SAL-biofilm inhibition may be due to the chelating action of SAL, but the authors did not investigate further this hypothesis. On the other hand, different iron chelating molecules inhibit the formation of *S. aureus* biofilms by interference of the ionic attractive forces established among the different matrix components (Ardehali et al., 2002; Lin et al., 2012). Exposure of *S. aureus*, NCTC8325, which is *rsbU* defective, to different sulfhydryl compounds resulted in diminution of biofilm formation by limiting PIA biosynthesis probably through metabolic interventions (Wu et al., 2011). Indeed, SAL has been shown to reduce biofilm formation by staphylococci through not well understood mechanisms (Teichberg et al., 1993; Prithiviraj et al., 2005). In the present study, the increment of maximum thickness and biomass was detected in biofilms grown in the presence of SAL by CLSM analysis and also by spectrophotometric assessment. It was found that the addition of iron returned the biofilm features to values similar to those of the controls. Our findings are consistent with those of Johnson et al. (2005) who reported that *S. aureus* biofilm production was induced under iron-restricted conditions. However, the authors did not observe a significant increment of PIA as the biofilm component induced under iron-depletion conditions. These discrepancies may be due to the level of iron concentration present in the different culture media used to develop biofilms. The authors utilized the minimal medium Chelex 100 resin-treated RPMI (it does not contain iron) to grow the *S. aureus* biofilm, and here we used the nutritive medium TSB (it contains 39.3 μM of iron basal) supplemented with 2 mM of SAL (~23% of reduction of medium iron concentration). Bacteria within biofilm respond to iron in a range narrower than that of the planktonic cells (Weinberg, 2004). Therefore, SAL would represent an environmental stress factor to bacteria that acts by modification of the iron levels thus contributing to increase the biomass by augmentation of PIA expression in biofilms. External signals such as NaCl or ethanol added to the culture medium also activated the staphylococci *ica* operon (O'Gara, 2007).

In this investigation, a relatively high concentration (2 mM) of SAL was chosen bearing in mind that it is a therapeutic concentration of aspirin to treat inflammatory diseases (Laudy et al., 2016). As a matter of fact, the effect of biofilm induction was also observed in MRSA and MSSA strains grown with 0.36 mM of SAL, a concentration similar to that found in sera of individuals treated with low doses of aspirin daily to prevent thrombosis events (see Figure 3). It can be speculated that the presence of SAL in biologic fluids may contribute to create microenvironments more restricted in free iron contents at the infection site forcing *S. aureus* to intensely compete with the host for this essential nutrient. As a partial evidence of this, we demonstrated that *S. aureus* grown in the presence of SAL provoked the release of iron from human transferrin by diminishing the pH through an increased production of lactate in biofilms. It is important to highlight that different *S. aureus* strains could respond dissimilarly to available iron. In this regard, the production of biofilms by several *S. aureus* strains related to CC8/USA300 or CC5/USA100 lineages was increased (1.25–3.87 times) in the presence of SAL, and that the addition of iron significantly reverted these increases. These results reinforce the observed effect of SAL, which is independent of the strain involved, methicillin susceptibility status or clonal genomic characteristics (CC8 or CC5).

FTIR techniques have been used previously to probe the presence of CP (Grunert et al., 2013) in order to characterize the staphylococcal surface glycopolymer composition (Johler et al., 2016) and also to identify the extracellular constituents of biofilms (Karadenizli et al., 2007). By PCA of the FTIR spectra using the spectral range specific for polysaccharides (1200–800 cm^−1^), it was possible to interpret the quantitative and qualitative variations of the surface glycopolymers of *S. aureus* in biofilm and planktonic lifestyles under three different culture conditions. This analysis showed that both planktonic and sessile cells grown in the presence of SAL clustered separately from their respective control counterparts in the PCA scattergram. However, iron addition promoted changes toward the zone of the control group only in those cells growing in biofilm lifestyle. These findings showed that FTIR data provide spectroscopic evidence that SAL modifies the polysaccharide features of *S. aureus* growing in one or the other lifestyle, although only in the biofilm lifestyle modifications in the polysaccharides become apparent when iron concentrations are diminished by SAL. Conversely, the polysaccharide perturbations detected by FTIR analysis of SAL-induced biofilms formed by the *codY S. aureus* mutant remained unchanged when iron was added to the medium.

*S. aureus* can produce either PIA or CP from the same biosynthetic precursor (UDP-*N*-acetylglucosamine) during the exponential and post-exponential growth phases, respectively (Sadykov et al., 2010a). We have previously described that SAL reduced the expression of CP in *S. aureus* grown in planktonic lifestyle (Alvarez et al., 2010). In the present study, however, *S. aureus* forming biofilms did not produced CP in any of the conditions under study suggesting that the polysaccharide perturbations analyzed by FTIR spectroscopy may correspond to PIA, the other major polysaccharide produced by *S. aureus*. More important, quantitative evaluation demonstrated that PIA plays a major role in extracellular matrices of biofilms formed by Newman, CBS and BRZ strains in the presence of SAL. It is generally accepted that MRSA strains develop biofilm by a PIA-independent mechanism (McCarthy et al., 2015). In particular, the BRZ lineage displays increased ability to accumulate *ica*-independent biofilm (Costa et al., 2013). It is worth noting that increases in the PIA concentration due to the action of SAL on MRSA strains (both CBS and BRZ) resembled that of the MSSA (Newman) strains indicating that SAL induces PIA-mediated biofilms by *S. aureus* independently of the methicillin susceptibility status under the conditions studied. On the other hand, these results suggest that the increased synthesis of PIA in the presence of SAL may contribute to build biofilms more tolerant to the action of antibiotics and host's defenses. Thomas et al. (2013) determined that a dysfunctional TCA cycle makes *S. epidermidis* less susceptible to beta-lactam antibiotics. Indeed, *S. epidermidis* can develop a PIA-dependent biofilm which is promoted by a reduced TCA cycle activity (Sadykov et al., 2010b).

The TCA cycle possess iron-sulfur cluster-containing proteins, such as the aconitase CitB, which is involved in the conversion of citrate to isocitrate. In the present study, SAL markedly decreased the transcription of *citB* in mature (24 h of growth) biofilms. Indeed, the finding that low levels of *citB* transcripts in *S. aureus* forming biofilms when compared with cells in planktonic lifestyle has been reported previously (Beenken et al., 2004). Moreover, a significant decrease of *citB* transcripts by the Newman strain grown in iron-depleted liquid media was reported previously (Friedman et al., 2006). The same authors reported that *citB* is positively regulated by Fur and iron in the *S. aureus* Newman strain. This observation correlates with the diminution of *citB* transcripts observed in biofilms grown with SAL during 24 h and it is in accordance with previous findings (Friedman et al., 2006). In fact, the *fur* transcription is initiated when the iron content diminishes as a result of its autoregulatory mechanism (Carpenter et al., 2009). The presence of SAL decreased the intracellular iron contents in bacteria forming biofilm by 24 h promoting *fur* up-regulation under this iron-limited condition. In concordance with the transcriptional data from biofilms, the enzymatic activity of aconitase was diminished in the presence of SAL, a finding that also indicates a reduction of the TCA cycle activity. In accordance with other authors, the altered TCA cycle activity due to the presence of SAL does not produce the intermediates required for CP biosynthesis in the sessile cells (Sadykov et al., 2010a).

It was recently hypothesized that, in staphylococci, any environmental signal or regulators capable of altering the TCA cycle activity may transform the metabolic status of bacteria thus resulting in the expression of genes required for growth in the altered environment (Richardson et al., 2015). Similarly, the low TCA cycle activity induced by SAL provoked the redirection of the central metabolism of the cells forming biofilm toward the fermentative pathway, enhancing Ldh expression and therefore stimulating lactate production. The presence of SAL during biofilm formation by *S. aureus* produced high levels of lactate thus favoring the drop of the extracellular pH and promoting the release of iron from human transferrin in order to compensate the diminution of free iron by SAL. Growth of the Newman strain in the presence of SAL reduced the culture media pH to values below 5.4, a level very favorable for the acquisition of iron by *S. aureus* (Cohen et al., 1967).

The results obtained in the present study suggest that SAL stimulates only the lactic fermentation pathway, since the levels of acetate were undetectable. It is likely that the *2*,*3*-butanodiol pathway may also be induced by SAL due to the increment of the enzyme acetoin reductase (ButA), which may explain the lack of extracellular acetate. Sadykov et al. (2008) reported that the *S. epidermidis* TCA cycle inactivation resulted in a derepression of the PIA biosynthesis genes and a redirection of carbon from cell growth into PIA biosynthesis. In the present work, the change on the carbon flow to the lactic fermentative metabolism of *S. aureus* caused a high level of *glmM* transcripts in immature biofilms grown in the presence of SAL, which may lead to an increase of the UDP-glucosamine precursor and, therefore, to the increment of PIA instead of CP production in SAL-induced biofilms. Other evidences regarding the metabolic status of *S. aureus* in SAL-induced biofilms are the decrease of *gapB* expression and the increase of *pykA* expression in mature biofilms grown in the presence of SAL. Unlike planktonic cells, bacteria forming biofilm constitute a heterogeneous population and, therefore, it is reasonable to find low transcriptional levels in general in bacteria adopting the biofilm lifestyle.

The TCA cycle is controlled by several transcriptional factors (e.g., Fur, CodY, among others) which respond to the intracellular concentration of metabolites (Geiger and Wolz, 2014). It should be noted that both the *cap* and *ica* operons contain binding sites for CodY and that CodY downregulates the expression of these operons (Majerczyk et al., 2008, 2010; Thoendel et al., 2011). Our results obtained from experiments performed on the Newman background are in agreement with findings previously reported by others. In this regard, SAL downregulated *codY* transcription thus releasing the repression of the *ica* locus by CodY. Interestingly, the addition of iron did not reverse this situation showing that iron positively affects the CodY protein (Friedman et al., 2006). Unlike other bacterial species, the *S. aureus codY* mutant exhibited a diminished ability to form biofilm (Richardson et al., 2015). We demonstrated here that mutation of *codY* diminished the ability of *S. aureus* to form biofilm but, the inductor effect of SAL on the biofilm biomass was also observed in the *codY* mutant. Unlike the Newman strain, however, its *codY* mutant was unable to reverse the SAL effect on biofilm formation after the addition of iron. Moreover, the biofilms formed by the *S. aureus codY* mutant exhibited a higher level of PIA production when compared with those developed by the Newman strain. One suitable explanation for these observations is that both metabolic and regulatory (low TCA cycle activity and downregulation of *codY*) changes generated by SAL may lead to enhanced PIA production. It is likely that the absence of CodY expression in the mutant was supported by the metabolic change leading to similar results in both the *codY* mutant and the wild-type strain. In contrast, the *codY* mutant was unable to reverse the SAL effect on biofilm formation and also failed to reverse the enhanced PIA production mediated by SAL after the addition of iron when compared with the wild-type strain. Friedman et al. (2006) showed that the CodY protein expression is stimulated by iron in the Newman strain. In this regard, our results suggest that CodY is involved in the decrease of the enhanced biofilm mediated by iron and this may be attributed to its regulatory effect on PIA synthesis.

*S. aureus* is able to colonize the murine nasal tissue forming a robust biofilm with an extensive extracellular matrix (Reddinger et al., 2016). Moreover, Lin et al. (2015) reported a significant contribution of PIA to the adherence of *S. aureus* to nasal epithelial cells. Additionally, nasal colonization with the Newman strain was registered for long periods in a model of nasal colonization using humanized transgenic mice (Xu et al., 2015). In the present work, administration of SAL to mice by the intravenous route increased the level of *S. aureus* nasal colonization. *S. aureus* can be found living asymptomatically in the human nasal vestibule of ~20% of the human population (Mulcahy and McLoughlin, 2016). If these individuals have a significant SAL concentration in serum due to aspirin consumption or vegetarian diet, it could be speculated that not only *S. aureus* colonization may endure, but also that eradication of this species from colonization sites may be hampered, thus increasing the risk of endogenous infection (Brown et al., 2014).

## Conclusion

Taken together, our data demonstrate that the presence of SAL, the active component of aspirin, which exhibits moderate iron-chelating capacity, strongly promotes *S. aureus* biofilm production in a PIA-dependent manner (Figure S4). These effects are the consequence of: (i) the induction of changes in the metabolic status of bacteria forming biofilm, such as low TCA activity and high lactate production (fermentative pathway preference) that provokes a diminution of the external pH level thus promoting the release of iron from human transferrin and the consequent acquisition of this essential nutrient; and (ii) the reduction of *codY* transcription and the iron concentration decrease provoked by SAL diminished the iron effect on CodY (Friedman et al., 2006). The augmentation of PIA by SAL would be responsible of the high asymptomatic nasal colonization in mice. Indeed, the increase in *S. aureu*s biofilm production induced by SAL may contribute to infection persistence.

## Author contributions

Conceived and designed the experiments: CD and FB. Performed the experiments and analyzed the data: CD, TG, AL, MB. Contributed the CLSM analysis tools: NC, OY. Wrote the manuscript: CD, FB. Revised the work critically: TG, DS, ME. Procured funding: TG, ME, DS, FB. All authors read and approved the final manuscript.

## Funding

This research was supported by grants from CONICET PIP 1122015010031CO (FB) and UBACyT: 20020150100126BA (FB) and 20020130100331BA (DS), ANPCyT (PICT 2014-0941) (DS), BMWF/MINCyT Bilateral Cooperation Program AR06/2013 (ME, FB) and start-up project, Vetmeduni Vienna (TG).

### Conflict of interest statement

The authors declare that the research was conducted in the absence of any commercial or financial relationships that could be construed as a potential conflict of interest.
