# Supplementary material for: The Active Component of Aspirin, Salicylic Acid, Promotes Staphylococcus aureus Biofilm Formation in a PIA-dependent Manner
Source: Front Microbiol. 2017 Jan 23;8:4. doi: 10.3389/fmicb.2017.00004 (PMC5253544; doi:10.3389/fmicb.2017.00004)
Supplement: Supplementary file 1 [file DataSheet1.DOCX]

**SUPPLEMENTARY MATERIAL**


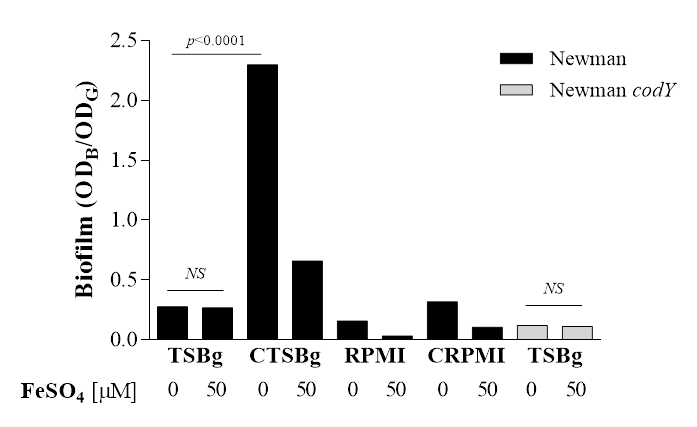


**Figure S1: Biofilm formation by the Newman strain and the *codY* mutant in different media of growth.** Biofilms were formed in the indicated media during 24 h in the presence or absence of 50 µM of FeSO_4_. Each bar represents the median from 2-3 independent experiments in sixtuplicate of samples. The biofilms were quantified by crystal violet staining (OD_B_) and expressed relative to the final culture density (OD_G_). Comparisons are represented by lines and each *p* values are denoted above. CTSBg and CRPMI represent Chelex 100 resin-treated TSBg and RPMI, respectively.

**Figure S2: Metabolically active *S. aureus* cells forming biofilm as determined by the MTT assay.** Biofilms were formed in TSBg during 24 h under in the conditions indicated. Metabolic activity was determined using the MTT (3-(4, 5-dimethylthiazol-2-yl)-2, 5-diphenyl tetrazolium bromide) assay according to Grare et al. (2008). An aliquot of MTT (1 mg/ml) was added to the wells and the plate incubated for 1 h. After the supernatants were discarded, the biofilms were washed with PBS, dried and dimethyl sulfoxide was added. The optical density of the wells was measured at 570 nm (OD_570_) was measured using an ELx800 Universal Microplate Reader. Comparisons among groups (without iron vs. with iron) were tested by the Mann-Whitney test. Each bar represents the mean ± SD.

**Reference:**

Grare, M., Fontanay, S., Cornil, C., Finance, C., and Duval, R. E.: (2008) Tetrazolium salts for MIC determination in microplates: Why? Which salt to select? How? *J. Microbiol. Methods.* 75,156–159.

**
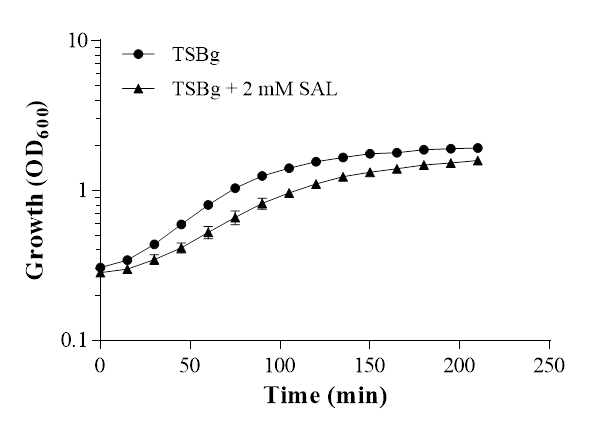
**

**Figure S3: Growth curve of the Newman strain cultured growth in the presence of SAL.** Culture of the Newman strain in TSBg in the presence or absence of 2 mM of SAL was monitoring at different times by through OD_600_ determination. Each dot represents the mean ± SEM of 3 independent experiments.

**Figure S4: Schematic representation of SAL effects on biofilm formed by *S. aureus*.** Summary of changes determined in mRNA expression, biochemical and phenotypic levels. Red circles and yellow hexagons denote iron and SAL molecules, respectively. Up and down yellow arrows represent an increased or diminished effect induced by SAL, respectively.

**Table S1: List of primers used for quantitative RT-PCR.**

| **Gene** | **Primer sequence** |
| --- | --- |
| *gyrB* | F: *5´GGTGCTGGGCAAATACAAGT*  R: *5´TGGGATACCACGTCCGTTAT* |
| *citB* | F: 5´*TCAAAATCCATCATTCTTCC*  R: *5´TCACCGAATTTACCCATAAC* |
| *fur* | F: *5´GACAGTATACAGAACGTTAGAG*  R: *5´GACCACATTCCATACATAC* |
| *pykA* | F: *5´TGCAGCAAGTTTCGTACGTC*  R: *5´GGGATTTCAACACCCATGTC* |
| *gapB* | F: *5´GCATTATTCCTACTTCTACTGG*  R: *5´GCTTGGTTTACTTCTTCTGC* |
| *ldh1* | F: *5´TTCGAGGAGATGTTATGG*  R: *5´ACAGATGACAACTAGATCC* |
| *glmM* | F: *5´CTAGTGATGATGTTGAACC*  R: *5´CAAGAAAACCCAGAATTACC* |
| *codY* | F: *5´TAGCACAAACGATTAGTAGC*  R: *5´CAGCATATTCACCTAGTACC* |
| *icaA* | F: *5´GCCATGTGTTGGATGTTGGT*  R: *5´AACCTTTTCGTTTTCATTGTGCT* |
| *cap5K* | F: *5´CCAGTGAATTGTTTGCAACG*  R: *5´CATTTTCCCAATAAATGTTGAAAG* |
